# Vaccine-elicited memory CD4^+^ T cell expansion is impaired in the lungs during tuberculosis

**DOI:** 10.1371/journal.ppat.1006704

**Published:** 2017-11-27

**Authors:** Stephen M. Carpenter, Jason D. Yang, Jinhee Lee, Palmira Barreira-Silva, Samuel M. Behar

**Affiliations:** 1 Department of Microbiology and Physiological Systems, University of Massachusetts Medical School, Worcester, Massachusetts, United States of America; 2 Division of Infectious Disease, Department of Medicine, Brigham and Women’s Hospital, Boston, MA, United States of America; 3 Division of Infectious Disease and Immunology, Department of Medicine, University of Massachusetts Medical School, Worcester, MA, United States of America; 4 Graduate School of Biomedical Sciences, University of Massachusetts Medical School, Worcester, Massachusetts, United States of America; New Jersey Medical School, UNITED STATES

## Abstract

Immunological memory is the key biological process that makes vaccines possible. Although tuberculosis vaccines elicit protective immunity in animals, few provide durable protection. To understand why protection is transient, we evaluated the ability of memory CD4^+^ T cells to expand, differentiate, and control *Mycobacterium tuberculosis*. Both naïve and memory CD4^+^ T cells initially proliferated exponentially, and the accumulation of memory T cells in the lung correlated with early bacterial control. However, later during infection, memory CD4^+^ T cell proliferation was curtailed and no protection was observed. We show that memory CD4^+^ T cells are first activated in the LN and their recruitment to the lung attenuates bacterial growth. However, their interaction with Mtb-infected macrophages does not promote continued proliferation. We conclude that a lack of sustained expansion by memory-derived T cells in the lung limits the durability of their protection, linking their slower expansion with transient protection in vaccinated mice.

## Author summary

Vaccines elicit pathogen-specific memory T cells whose early and potent activation upon infection should provide long-lasting control of bacterial growth. Although many experimental vaccines generate memory CD4^+^ T cells and can control the growth of *Mycobacterium tuberculosis* (Mtb) early during infection, none reliably provide protection from pulmonary tuberculosis (TB) that is durable. Although the etiology of the clinical failure of memory T cells is not well understood, few studies monitor memory T cell fate and function throughout chronic infection. Using both clonal and polyclonal models of Mtb-specific memory CD4^+^ T cell function during TB, we show that the expansion of memory-derived T cell responses is impaired in the lungs, compared with the primary (naïve) CD4 response. Despite expressing a protective effector phenotype, and reducing bacterial growth early after Mtb challenge, we further show that memory CD4^+^ T cells do not proliferate in response to Mtb-infected macrophages. Their impaired expansion corresponded with waning protection in vaccinated mice later during infection. We propose that both the induction of memory T cell proliferation by infected macrophages, and the durability of vaccine-elicited T cell responses during TB should serve as preclinical vaccine benchmarks.

## Introduction

A vaccine that prevents active pulmonary tuberculosis (TB) is urgently needed to reduce *Mycobacterium tuberculosis* (Mtb) transmission and TB incidence. M. bovis-*BCG* (BCG) is the only currently approved TB vaccine. However, neither BCG nor MVA85A, the only other TB vaccine to complete an efficacy trial, reliably prevent active pulmonary disease [1,2]. While evidence of TB prevention in BCG-vaccinated populations has been shown in temperate climates, protection is notably short-lived, waning after adolescence [1]. Furthermore, Individuals successfully treated for active pulmonary TB are not protected from further episodes of TB attributable to reinfection [3]. The inability of vaccination, or prior infection, to reliably reduce TB incidence indicates the need for a better understanding of why memory T cells do not function more effectively after Mtb challenge.

Vaccination mimics pathogen-elicited memory, and has fundamentally changed our ability to prevent infectious diseases. Although most clinically efficacious vaccines work by eliciting memory B cells and antibodies, vaccines that elicit T cell memory work by increasing the frequency, affinity, and function of microbe-specific T cells compared to their counterparts in the naïve T cell repertoire [4]. However, the requirements for protective immunity against slowly-progressive infection appear to be different than those for acute viral infection, where viral replication is rapid and antigen is abundant. For chronic infections, such as TB, memory T cells must not only respond rapidly, but their effect must be long-lasting. Accordingly, TB vaccines show little evidence of long-lived protection in mice despite reducing bacterial burden 5–10 fold early during infection [5–7]. Prior pulmonary TB in mice also leads to an early, 10-fold reduction in bacterial growth upon reinfection [8], but does not confer a long-term survival benefit [5]. These data suggest an early but transient benefit of memory T cells against Mtb.

CD4^+^ T cells play a dominant role in host defense during active pulmonary TB [9]. CD4^+^ T cells attenuate Mtb growth by direct recognition of infected cells through TCR recognition, acting via both IFNγ-dependent and independent effector functions [10–12]. A major limitation to T cell-mediated protection is the significant delay in their response to Mtb in the lungs. The mouse model of TB has revealed that this nearly 2 week delay is caused by the requirement for T cell priming to occur in the lung-draining mediastinal lymph nodes (MLN), which occurs 10–12 days after inoculation [13–15]. The recruitment of memory CD4^+^ T cells to the lung after Mtb challenge is similarly delayed [8,16], but whether memory CD4^+^ T cells are first activated in MLN or the lung has not been determined.

ESAT6 (EsxA) and Ag85b are two immunogenic proteins secreted by Mtb that elicit CD4^+^ T cell responses and have been incorporated into TB vaccine candidates [17]. However, these proteins differ in their levels of expression, function, and antigenicity during TB. While ESAT6 is a virulence factor expressed early and late during infection, Ag85b expression is dispensable for Mtb virulence, and its expression is downregulated *in vivo* as early as 3 weeks post-infection (wpi) [18,19]. Despite these differences, CD4^+^ T cell responses targeting either ESAT6 or Ag85b expand and reduce bacterial load early after Mtb challenge [20,21]. However, maintenance of both the numbers of memory-derived T cell responses and control of Mtb growth in vaccinated animals is rarely assessed at late time points.

In this study, we ask whether the fate of memory CD4^+^ T cells that respond to Mtb infection affects the protection conferred by vaccination. We observed that both ESAT6 and Ag85b vaccinations elicited memory CD4^+^ T cells that expanded and reduced bacterial load early after Mtb challenge. However, protection waned at late time points, as did the number of memory-derived CD4^+^ effector T cells in the lungs. Thus, the durability of the protection conferred by memory CD4^+^ T cells is linked to their potential for sustainable expansion, but we further observed that their proliferation was not promoted by their response to Mtb-infected macrophages. We speculate that vaccines which elicit memory T cells capable of sustainably expanding in response to infected cells will more durably control Mtb growth in the lung; a factor that should be considered in TB vaccine development.

## Results

### The augmented antigen-specific CD4^+^ T cell response in vaccinated mice is not durable after Mtb challenge

**Fig 1:**
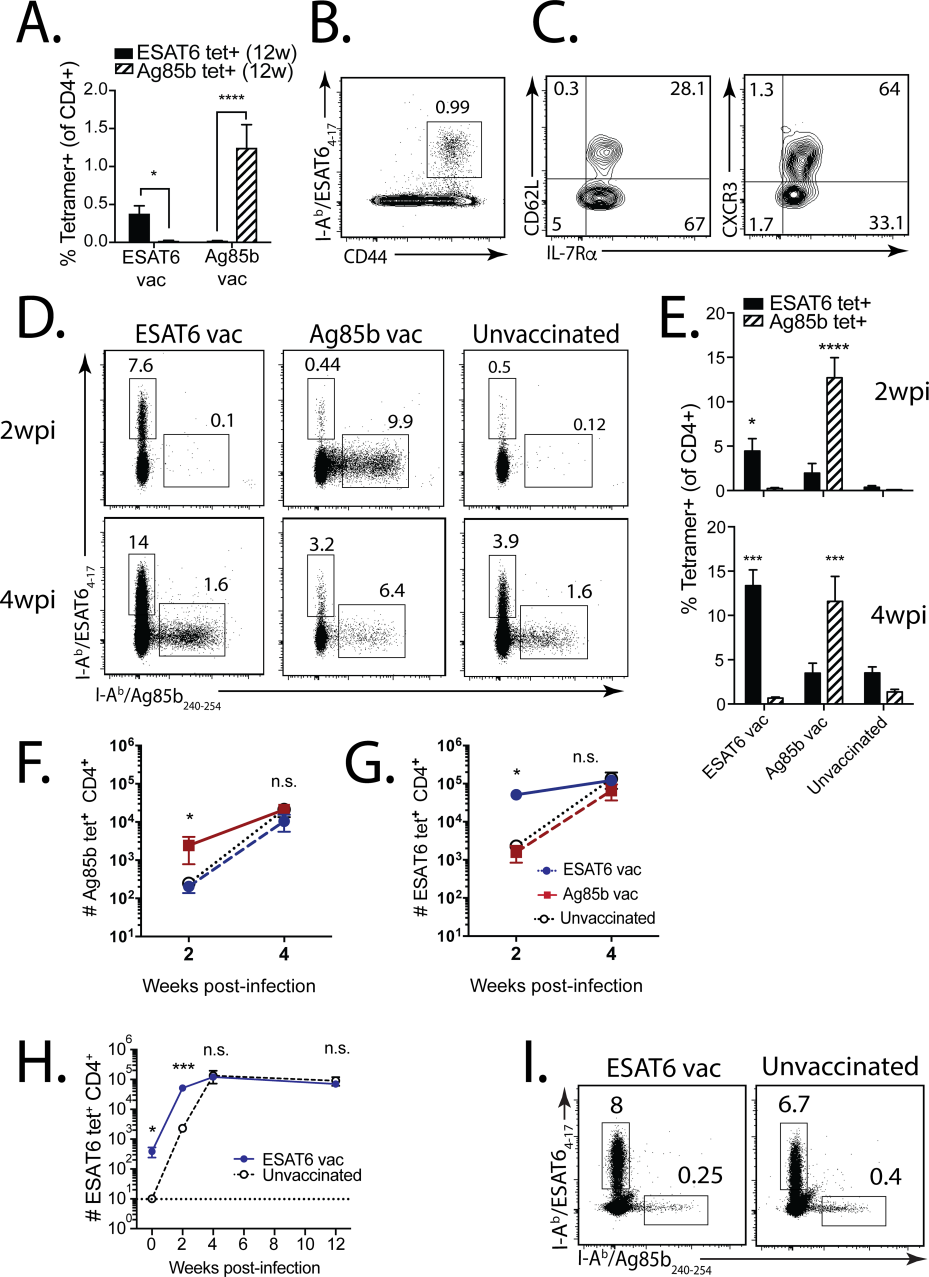
The augmented antigen-specific CD4^+^ T cell response in vaccinated mice is not durable after Mtb challenge. **(a)** ESAT6_4-17_ and Ag85b_240-254_ tetramer^+^ T cell responses in the blood 12 weeks after ESAT6_4-17_ or Ag85b_240-254_ s.c. peptide vaccination. **(b)** Representative ESAT6_4-17_ tetramer responses in the lung 12 weeks after ESAT6 vaccination, and **(c)** CD62L (left), CXCR3 (right), and IL-7Rα expression by these tetramer^+^CD4^+^ T cells. **(d)** Representative ESAT6_4-17_ and Ag85b_240-254_ tetramer responses in the lung 2 weeks (top) and 4 weeks (bottom) after Mtb infection of ESAT6-vaccinated (left), Ag85b-vaccinated (middle), or unvaccinated mice (right). **(e)** Compiled ESAT6- and Ag85b-specific responses at 2 weeks (top) and 4 weeks (bottom) post-infection (wpi) from vaccinated vs. unvaccinated mice. **(f)** Frequency of Ag85b-specific (left) and ESAT6-specfic (right) CD4^+^ T cells for each vaccine group, and unvaccinated, at 2 and 4 wpi. **(g)** ESAT6-specific CD4^+^ T cells for ESAT6-vaccinated vs. unvaccinated mice at 0, 2, 4, and 12 wpi. **(h)** ESAT6_4-17_ and Ag85b_240-254_ tetramer responses in the lungs of ESAT6-vaccinated (left) vs. unvaccinated (right) mice 12 wpi. In all figures, numbers in quadrants or gated regions of FACS plots represent percent events. * p<0.05, ** p<0.01, *** p<0.001, **** p<0.0001, n.s., not significant.

To determine whether vaccination is able elicit memory CD4^+^ T cells that establish a durable response and confer protection against Mtb challenge, we vaccinated mice against either ESAT6_3-17_ or Ag85b_240-254_, using a strategy previously shown to elicit protective ESAT6-specific CD4^+^ T cells [20]. We enumerated ESAT6_4-17_ and Ag85b_240-254_–specific responses after a single vaccination, prior to Mtb challenge. In the blood, 0.5–2% of CD4^+^ T cells were found to be specific for ESAT6 or Ag85b (Fig 1A). Memory CD4^+^ T cells were also identified in the lungs 12 weeks after ESAT6 vaccination (Fig 1B), consisting of both central (CD62L^Hi^ IL-7Rα^Hi^) and effector (CD62L^Lo^ IL-7Rα^Hi^) memory CD4^+^ T cells (Fig 1C). The majority of these memory CD4^+^ T cells expressed CXCR3, a chemokine receptor associated with trafficking of memory T cells to the airway during inflammation [22] (Fig 1C). These data indicate that vaccination elicited robust, long-lasting memory CD4^+^ T cell responses to two immunodominant Mtb antigens.

Twelve weeks after vaccination, we challenged vaccinated and control mice with aerosolized Mtb (strain Erdman). An early and robust ESAT6-specific CD4^+^ T cell response was detected 2 and 4 wpi in ESAT6 vaccinated mice (5–15% of CD4^+^), compared with Ag85b-vaccinated or unvaccinated mice (0.1–5% of CD4^+^) (Fig 1D and 1E). The Ag85b-specific response was similarly greater in Ag85b-vaccinated mice at these time points (10–15% vs. 0.1% of CD4^+^) (Fig 1D and 1E). Surprisingly, Ag85b- and ESAT6-specific CD4^+^ T cells accumulated more slowly in the lungs of vaccinated, compared with unvaccinated mice (Fig 1F and 1G). Although the proportion of Ag85b- or ESAT6-specific CD4^+^ T cells was usually greater in vaccinated mice at 4 wpi, we found no difference in the absolute numbers of antigen-specific CD4^+^ T cells (Fig 1F and 1G). To assess the durability of the memory response, we focused on the ESAT6 response since Ag85b is downregulated by Mtb by 3 wpi [18]. Although the proportion of ESAT6-specific CD4^+^ T cells in the lungs of ESAT6 vaccinated mice was initially greater (Fig 1D and 1E), there was no difference in the number or proportion of ESAT6-specific CD4^+^ T cells in the lungs of vaccinated vs. unvaccinated mice 12 wpi (Fig 1G and 1H). These data show an initial but transient response of memory CD4^+^ T cells during infection, similar to the response of memory CD8^+^ T cells [23,24].

### The primary and secondary Ag85b-specific CD4^+^ T cell responses use similar TCRs

**Fig 2:**
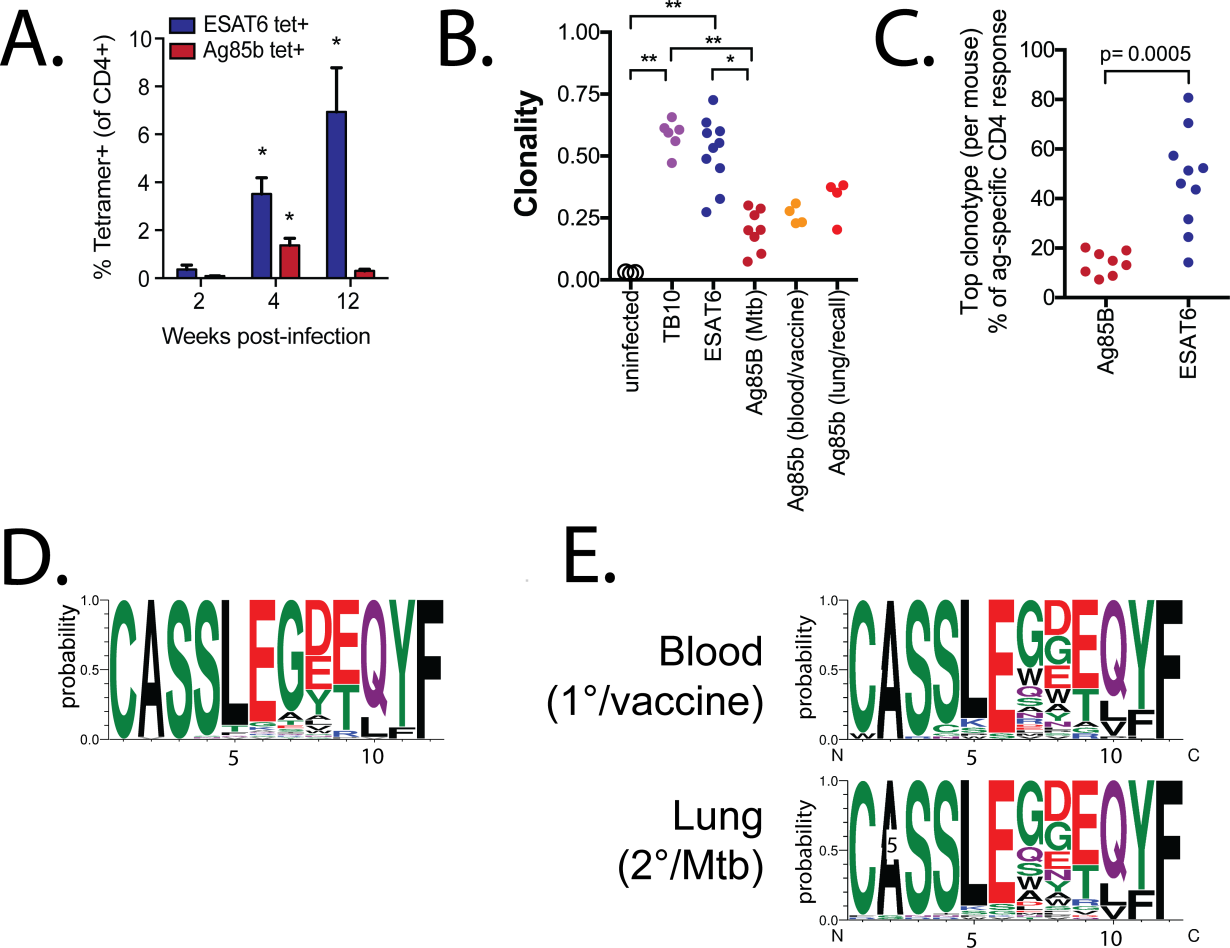
Primary and secondary Ag85b-specific CD4^+^ T cell responses use similar TCRs. **(a)** ESAT6- and Ag85b-specific CD4^+^ T cell responses in infected mice at 2, 4, and 12 wpi. **(b)** TCR clonality of splenic T cells from normal C57BL/6 mice (n = 3), TB10.4-specific CD8^+^ T cells (n = 6) (both from [25]), ESAT6-specfic (n = 10) and Ag85b-specific (n = 8) CD4^+^ T cells from lungs 4–10 wpi; from blood 1 week after Ag85b_240-254_ vaccination and in the same mice 3 wpi (n = 4). **(c)** The top clonotypes from the Ag85b- or ESAT6-specific response from each Mtb-infected mouse. **(d)** CDR3β motifs for primary Ag85b-specific responses derived from TCRβs using TRBV16 with a CDR3β length of 12. **(e)** CDR3β motifs for Ag85b-specific CD4^+^ T cells that use TRBV16 with a CDR3β length of 12 in blood 10 d after vaccination (top row), or in lung 3 wpi (bottom row).

An explanation for the difference between the kinetics of the primary and the secondary CD4^+^ T cell responses is that the TCRs elicited by vaccination differ from those elicited by infection [23,25]. Primary ESAT6_4-17_ and Ag85b_240-254_-specific CD4^+^ T cell responses were detected in the lung as early as 2 wpi (Fig 2A). By 4 wpi, 3–6% of CD4^+^ T cells in Mtb-infected mice were ESAT6-specific and this response continued to be dominant during chronic infection (12 wpi). In contrast, the Ag85b-specific response peaked 4 wpi and subsequently declined. The TCRβ clonality of ESAT6-specific CD4^+^ T cells was very high, similar to TB10.4-specific CD8^+^ T cells (Fig 2B). In contrast, the clonality of Ag85b-specific CD4^+^ T cells was only slightly greater than splenic T cells from uninfected mice. Among Ag85b-specific CD4^+^ T cells, the most abundant clonotype from each subject had a mean of 14%. In contrast, the dominant clonotypes for ESAT6-specific CD4^+^ T cells varied between 14–81% (Fig 2C, S1 Data). To determine the molecular basis for the differences in clonality, we analyzed the structure of the TCRβ repertoires.

The Ag85b-specific CD4^+^ T cells had a skewed CDR3β length distribution with a mode of 12 aa and preferential use of TRBV16 (TCR Vβ11) compared to splenic T cells from uninfected mice (S2A Data). These TCRs accounted for 28% of the productive TCRβ rearrangements from Ag85b-specific CD4^+^ T cells. We detected 82 unique rearrangements encoding 28 different CDR3βs, indicating convergent selection, which were enriched in the amino acids “LEG” (Fig 2D). Six CDR3βs were expanded in two or more mice, and two CDR3βs (“CASSLEGDEQYF” and “CASSLEGDTQYF”) were detected in all eight mice. Thus, the Ag85b-specific CD4^+^ T cell response was characterized by a ‘public’ TCRβ response, as the same TCR was shared between multiple individuals and selected based on structural features of the CDR3β sequence. In contrast, there was no preferential use of TCRVβ genes among the ESAT6-specific CD4^+^ T cell repertoire, indicating that the response to ESAT6 is dominated by clonal expansions of private TCRs (S2B Data).

We next analyzed the TCR repertoire of CD4^+^ T cells elicited by Ag85b_240-254_ vaccination. Vaccine-elicited Ag85b-specific CD4^+^ T cells were similar to those from infected mice, with a skewed TCRβ repertoire: 46% used TRBV16 with a CDR3β length of 12. (Fig 2E). The mice were rested for 12 weeks after vaccination, challenged with Mtb and analyzed 3 wpi. The TCRβ repertoire of the secondary response to Ag85b was similar to vaccine-elicited CD4^+^ T cells: 45% used TRBV16 with a CDR3β length of 12. Both the post-vaccination and post-challenge samples were enriched for the “LEG” motif (Fig 2E). These data show that the TCRβ repertoire of vaccine-elicited and Mtb-recalled Ag85b-specific CD4^+^ T cells was similar to naïve T cells that undergo expansion after primary infection.

## Memory CD4^+^ T cells require activation in the lung-draining MLN

We next assessed whether the impaired expansion of memory T cells could be explained by differences between the primary and secondary CD4^+^ T cell responses in the timing or the location of their activation (Fig 1). A barrier to addressing this question is that primary and secondary CD4^+^ T cell responses cannot be reliably distinguished in intact mice. Therefore, we compared the primary and secondary responses by generating memory and naïve Ag85b-specific CD4^+^ T cells using P25 TCR transgenic (Tg) CD4^+^ T cells [15,26]. Although the CDR3β used by the P25 cells was not detected among the Ag85b-specific CD4^+^ T cells we isolated from the lungs of Mtb-infected mice, it is encoded by TRBV16 [26] (S3 Data).

**Fig 3:**
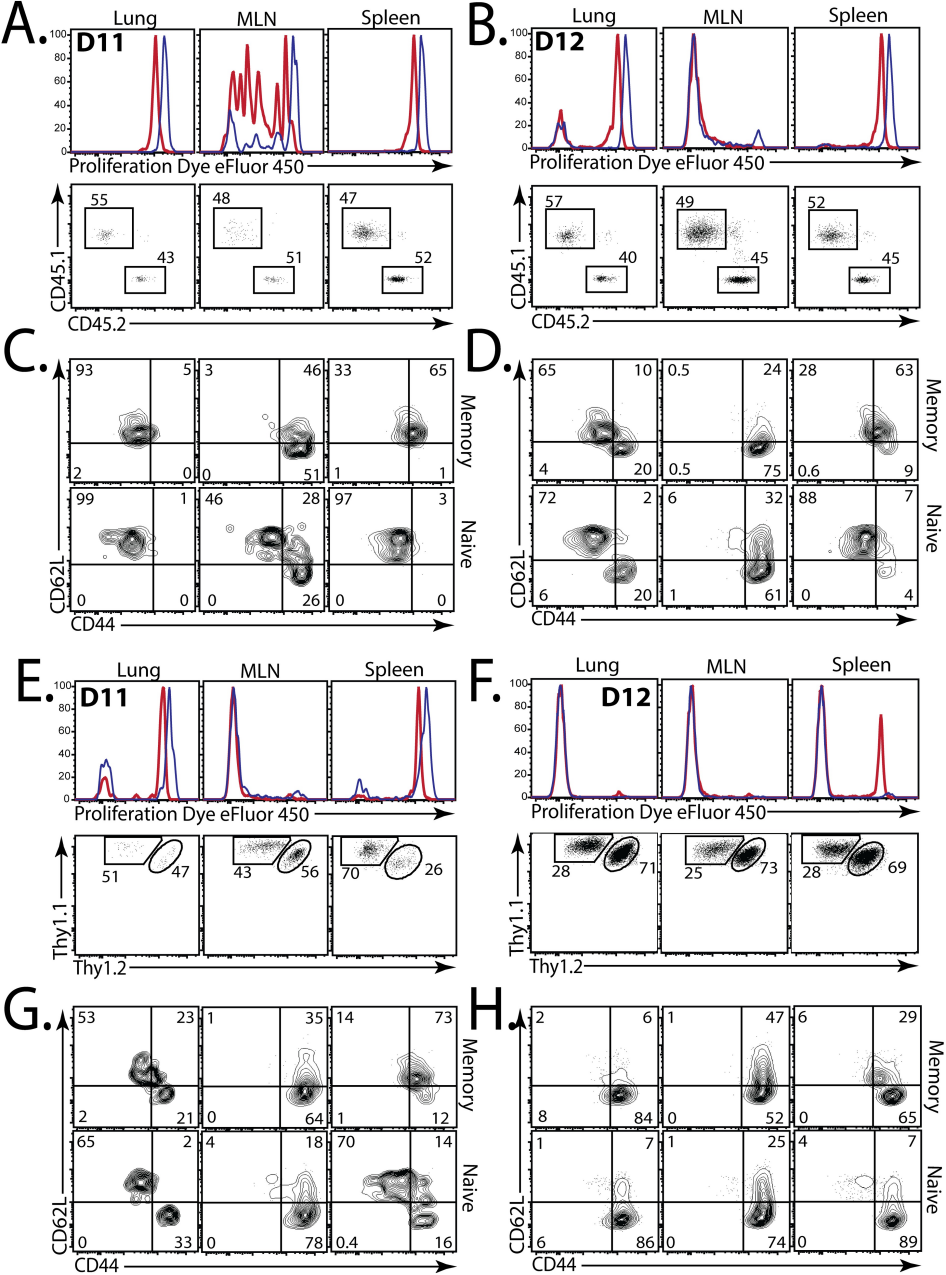
Memory CD4^+^ T cells are first activated in the MLN. **(a,b)** eFluor450 dilution (top), and the relative proportion (bottom) of memory-derived (red, CD45.1^+^) and naïve-derived (blue, CD45.2^+^) P25 Tg Ag85b-specific CD4^+^ T cells isolated from the lungs, LN, and spleen on **(a)** d11, and **(b)** d12, after aerosol Mtb challenge of mice that received a 1:1 mix of memory and naive P25 cells at d7. **(c, d)** CD62L and CD44 expression by memory-derived (top) or naive-derived (bottom) P25 cells, isolated from lung (1^st^ column), MLN (2^nd^ column), or spleen (3^rd^ column) on **(c)** d11, and **(d)** d12. **(e, f)** eFluor450 dilution (top), and the proportion (bottom) of memory-derived (red, Thy1.1^+/+^) and naive-derived (blue, Thy1.1^+^/1.2^+^) C7 Tg ESAT6-specific CD4^+^ T cells isolated from the lungs, MLN, or spleen on **(e)** d11, or **(f)** d12, after Mtb challenge of mice that received a 1:1 mix of memory and naive C7 cells at d7. **(g, h)** CD62L and CD44 expression by memory-derived (top) or naive-derived (bottom) C7 cells in these mice, isolated from lung (1^st^ column), MLN (2^nd^ column), and spleen (3^rd^ column) at **(g)** d11, or **(h)** d12. Data are concatenated plots from 4 mice/group.

Flow-sorted memory (CD45.1^+^ TCR Vβ11^+^ CD44^Hi^ KLRG1^Lo^) CD4^+^ P25 cells from Ag85b-vaccinated mice, and naïve (CD45.2^+^ TCR Vβ11^+^ CD44^Lo^) CD4^+^ P25 cells were co-transferred at a 1:1 ratio into Thy1.1^+^ mice infected 7 days earlier with Mtb, before T cell priming normally occurs [13,15,25]. Both memory and naïve P25 cells began proliferating first in the lung-draining MLN at d11 post-infection (Fig 3A, top), while maintaining their 1:1 input ratio (Fig 3A, bottom), before proliferation in lung or spleen. As these plots represent concatenated events from multiple mice, the frequency of eFluor450^lo^ events in the lung represents a very small fraction of the population, whereas the eFluor450^lo^ events in the MLN represents a much larger population of robustly dividing C7 T cells. By d12, both memory and naïve P25 cells maintained a 1:1 ratio in the MLN, and proliferating P25 cells were detected in the lung (Fig 3B; S4A Data, top row). On d11, CD62L was downregulated by dividing memory and naïve P25 cells in the MLN at d11, but its levels did not change on the non-dividing P25 cells in the lung (Fig 3C). These data indicate that the memory CD4^+^ T cells in the lung have not recognized antigen before their activation in the MLN. By d12, downregulation of CD62L and upregulation of CD44 were detected on most memory- and naïve-derived P25 cells in the MLN, as well as the proliferating CD4^+^ T cells in the lung (Fig 3D). As the downregulation of CD62L occurred only on dividing cells (S4B Data), we conclude that they were primed in the MLN prior to trafficking to the lung.

We next addressed whether memory CD4^+^ T cells specific for ESAT6 also require activation in the MLN. To answer this question, we used the C7 TCR Tg CD4^+^ T cells, which contain a TCR specific for ESAT6_3-17_ [27]. Although the C7 TCR CDR3β sequence was infrequently detected among the ESAT6-specific CD4^+^ T cells from Mtb-infected mice (S5 Data), its CDR3β sequence contained the motif “GGG,” which was common among ESAT6-specific CD4^+^ T cells in vivo. Thus, the C7 TCR was representative of ESAT6-specific CD4^+^ T cells during infection (S3 and S5 Data). Similar to the Ag85b-specific responses, we found that the proliferation of memory (Thy1.1^+/+^) and naïve (Thy1.1/2^+^) C7 cells in the MLN preceded their proliferation in the lung on d11 (Fig 3E, top), indicating that memory CD4^+^ T cells specific for ESAT6 also require activation first in the MLN. Like P25 cells, memory and naïve C7 cells maintained their 1:1 input ratio during early after activation (Fig 3E, bottom; S4A Data, bottom row). Interestingly, we observed proliferation for C7 cells in both the MLN and lung at d11, earlier than we observed for P25, indicating that ESAT6-specific T cells became activated slightly earlier than Ag85b-specific T cells during infection (Fig 3E). By d12, both memory and naïve C7 cells proliferated robustly in the MLN (Fig 3F, top), and C7 cells in the lung also appear to have undergone multiple rounds of cell division (Fig 3F, top), with a skewed ratio favoring the primary (naïve-derived) C7 cells (Fig 3F, bottom). Downregulation of CD62L and increased CD44 expression were also found to be similar in both memory and naïve C7 cells at d11 (Fig 3G) and d12 post-infection (Fig 3H). Again, downregulation of CD62L occurred only on the dividing cells (S4C Data), which were more numerous in the MLN at d11 (Fig 3E), compared with the lung. Taken together, we conclude that memory CD4^+^ T cells, like naïve cells, require activation in the MLN prior to expansion in the Mtb-infected lung.

### Memory CD4^+^ T cells are less fit than naive CD4^+^ T cells during TB

**Fig 4:**
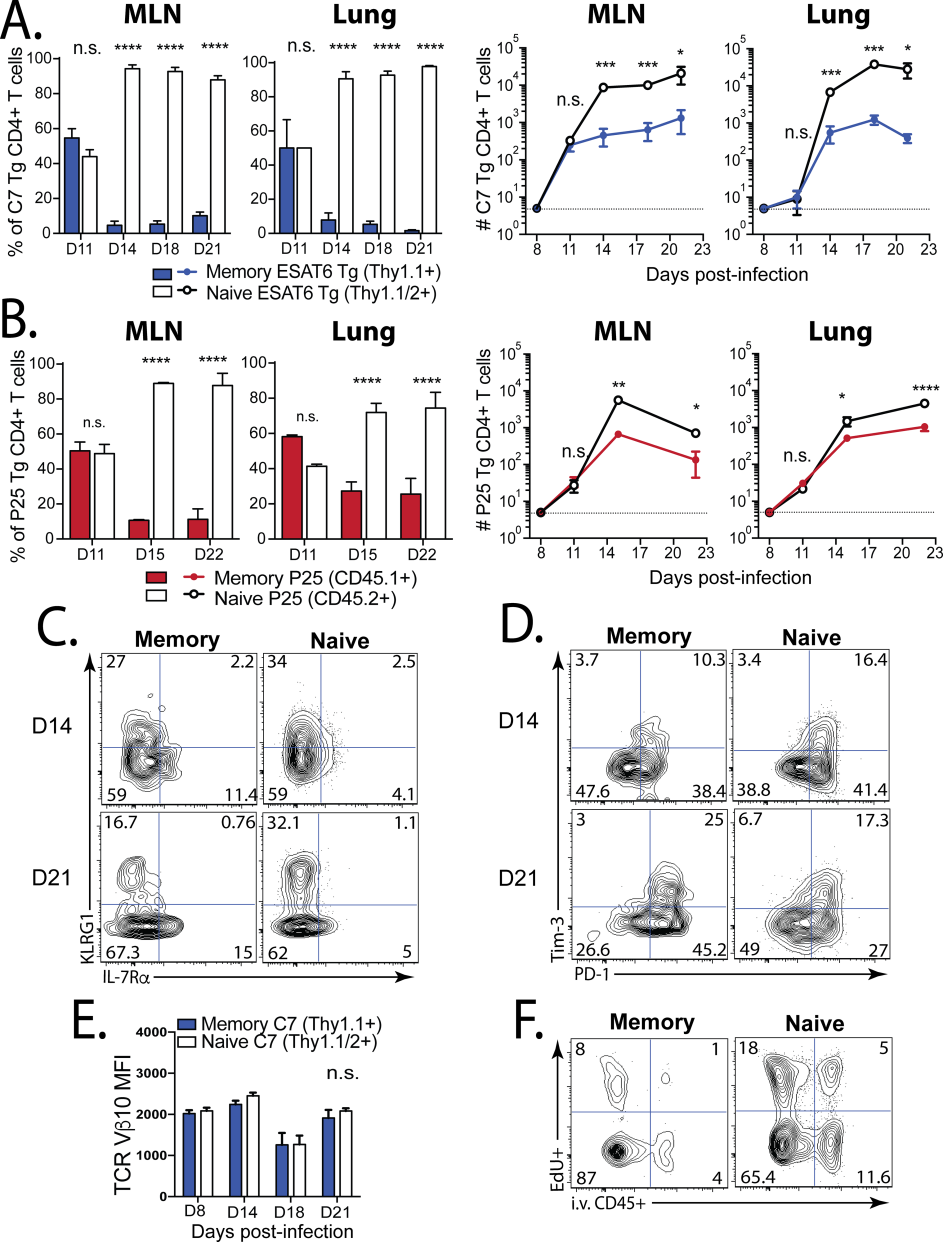
Memory CD4^+^ T cells are less fit than naive CD4^+^ T cells in TB. **(a)** A 1:1 mix of memory and naive C7 Tg ESAT6-specific CD4^+^ T cells were transferred into C57BL/6 mice on d7 post-infection. The ratios (left) and numbers (right) of memory-derived (blue) and naive-derived (white) C7 cells in the LN and lung were compared at the time points indicated. **(b)** A 1:1 mix of memory and naive P25 Tg Ag85b-specific CD4^+^ T cells were transferred into C57BL/6 mice on d7. The ratios (left) and cell numbers (right) of memory-derived (red) and naive-derived (white) P25 cells in the MLN and lung were compared. Representative plots of **(c)** KLRG1 and IL-7Rα expression. and **(d)** Tim-3 and PD-1 expression by the memory-derived (1^st^ column) and naive-derived (2^nd^ column) C7 cells (shown in ‘a’) in the lungs at d14 (top) and d21 (bottom) after Mtb challenge. **(e)** TCR Vβ10 expression by memory-derived (blue) vs. naive-derived (white) C7 cells from the lungs at the indicated time points after Mtb challenge. **(f)** Representative plots of EdU uptake and i.v. anti-CD45 binding by memory- (left) or naive-derived (right) C7 cells in the lung at d21 post-infection. Groups contained 4 mice each. MFI, mean fluorescence intensity.

We observed that the initial activation of memory and naïve CD4^+^ T cells in the MLN is similar (Fig 3). Therefore, we reasoned that any difference in the relative fitness of naïve and memory CD4^+^ T cells must manifest itself after their initial activation. Using the same adoptive co-transfer model in which naïve and memory T cells are compared during infection, we investigated the kinetics of T cell expansion after Mtb challenge. 1x10^4^ flow-sorted memory and naïve C7 CD4^+^ T cells were co-transferred at a 1:1 ratio into Mtb-infected mice. Both memory and naïve C7 cells initially expanded during priming in the lung-draining MLN 11 days post-infection (Fig 4A). However, by day 14, the memory-derived C7 Tg CD4^+^ T cells were outnumbered by the primary C7 response 20:1 in the MLN and lung (Fig 4A). The memory-derived response was similarly outnumbered even when the transferred cells were isolated without direct antibody-labeling or flow-sorting, which ruled out the possibility that our isolation strategy altered the function of the T cells (S6c Data).

To exclude the possibility that the limited capacity of memory CD4^+^ T cells to expand was limited to those specific for ESAT6, we used the same strategy with P25 Tg Ag85b-specific CD4^+^ T cells. Both memory and naïve P25 cells initially expanded in the MLN 11 days post-infection (Fig 4B). However, by day 15, the memory-derived P25 CD4^+^ T cells were outnumbered by the primary P25 CD4^+^ T cells 4:1 in the MLN and lung (Fig 4B). Both memory and naïve C7 and P25 CD4^+^ T cells differentiated into effectors T cells, most of which downregulated IL-7Rα, and some of which became terminally-differentiated based on KLRG1 expression (Fig 4C). No significant differences in the expression of KLRG1 or the inhibitory receptors PD-1 and Tim-3 were observed at d14 (Fig 4C and 4D) [28]. At d21, greater numbers of naïve-derived C7 cells expressed KLRG1, and slightly more of the memory-derived cells expressed PD-1, but not Tim-3 (Fig 4C and 4D). Furthermore, we did not detect differences in TCR expression at any time point (Fig 4E). Finally, the memory-derived C7 cells were more frequently located in the “parenchymal” lung compartment than the naive-derived C7 CD4^+^ T cells, based on the i.v. injection of labelled anti-CD45. However, fewer of the memory-derived cells were found to be proliferating (EdU+) (Fig 4F). These data indicate that memory-derived CD4^+^ T cells specific for ESAT6 do not expand as well as the primary ESAT6-specific response in the lungs during TB, despite an early response to Mtb challenge. Taken together, these data indicate that on a per cell basis, the expansion, but not the differentiation or trafficking, of memory CD4^+^ T cells specific for Ag85b and ESAT6 are impaired in the context of Mtb infection in the lungs.

### Memory and naïve CD4^+^ T cells have a similar activation threshold

**Fig 5:**
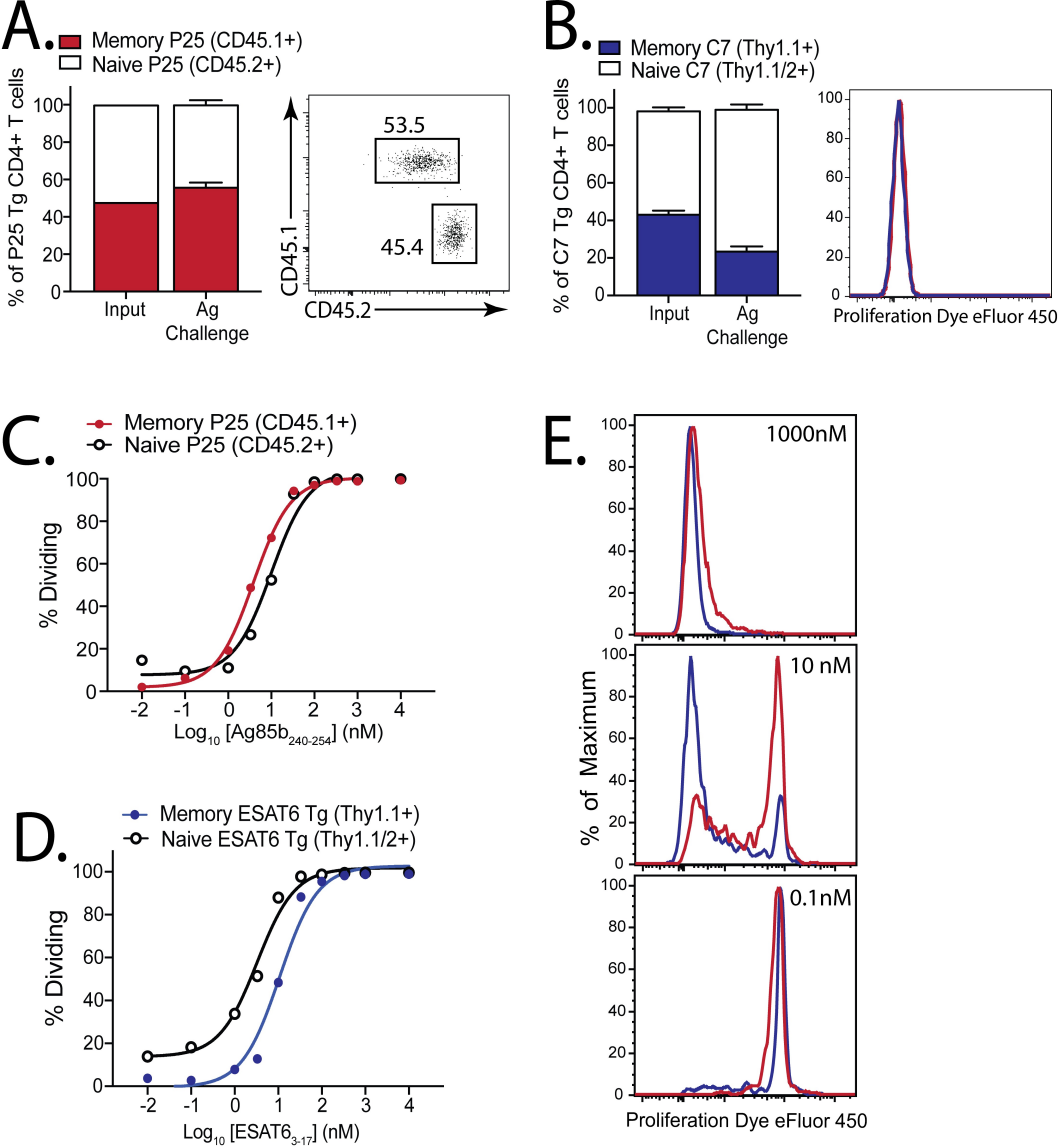
A similar antigen threshold is required to activate memory and naive CD4^+^ T cells. **(a)** Ratios of memory (red, CD45.1^+^) and naive (white, CD45.2^+^) P25 Tg CD4^+^ T cells 1 d after 1:1 co-transfer into congenically-marked mice (input ratio), or 3 d after challenge with Ag85b_240-254_/poly(I:C)/αCD40; representative d3 plot showing ratio (right). **(b)** Ratios of memory (blue, Thy1.1^+/+^) and naive (white, Thy1.1^+^/1.2^+^) C7 Tg CD4^+^ T cells 1 d after 1:1 co-transfer into congenically-marked mice (input), or 3 d after challenge with ESAT6_3-17_/poly(I:C)/αCD40; representative d3 plot showing eFluor450 dilution (right). Groups contained 4 mice each. Dose-response curves of proliferating naive or memory **(c)** P25, or **(d)** C7 cells, 3–4 d after culture with peptide-coated splenocytes. **(e)** Representative eFluor450 dilution by memory (red) and naive (blue) C7 cells exposed to different peptide concentrations.

We next asked whether the difference in T cell fitness is an intrinsic property or a consequence of the environment in the Mtb-infected lung. Memory and naïve P25 cells were transferred at a 1:1 ratio into uninfected mice, followed by vaccination with Ag85b_240-254_ peptide together with poly(I:C) and anti-CD40 mAb, which potently stimulates large CD4^+^ and CD8^+^ T cell responses [29,30]. After high-dose antigen challenge, both memory and naïve P25 cells robustly proliferated and maintained their input 1:1 ratio (Fig 5A). Memory ESAT6-specific C7 CD4^+^ T cells also proliferated after ESAT6_3-17_ challenge (Fig 5B), although their expansion was not quite as efficient as naïve C7 CD4^+^ T cells.

We recently showed that memory CD8^+^ T cells specific for TB10.4 have a higher activation threshold than naïve CD8^+^ T cells [23], a phenomenon originally described in an ovalbumin vaccination model [31]. To determine whether higher concentrations of antigen were required to activate memory vs. naïve CD4^+^ T cells, we cultured memory and naïve P25 cells with APCs loaded with different amounts of Ag85b_240-254_ peptide and measured their proliferation 72–90 hours later. Similar amounts of Ag85b_240-254_ peptide were required to induce memory and naïve P25 CD4^+^ T cell proliferation (Fig 5C). Next, we assessed whether memory and naïve C7 Tg ESAT6-specific CD4^+^ T cells have a similar activation threshold. The proliferation of a 1:1 mix of memory and naïve C7 cells was measured after stimulation with ESAT6_3-17_ peptide. In contrast, slightly more peptide was required to activate (CD25 expression) or induce proliferation of memory C7 CD4^+^ T cells compared to naïve C7 CD4^+^ T cells (Fig 5D and 5E). Interestingly, these results correlate with the behavior of the T cells in vivo (compare Fig 4A and 4B and Fig 5A and 5B). By using TCR Tg CD4^+^ T cells specific for two different Mtb antigens, we show that differences in activation threshold may modulate the expansion of memory CD4^+^ T cells in the lungs during Mtb infection, whereas memory CD4^+^ T cells are able to proliferate well after non-infectious challenge *in vivo*.

### Mtb-infected DCs, but not macrophages, stimulate memory CD4^+^ T cell proliferation

**Fig 6:**
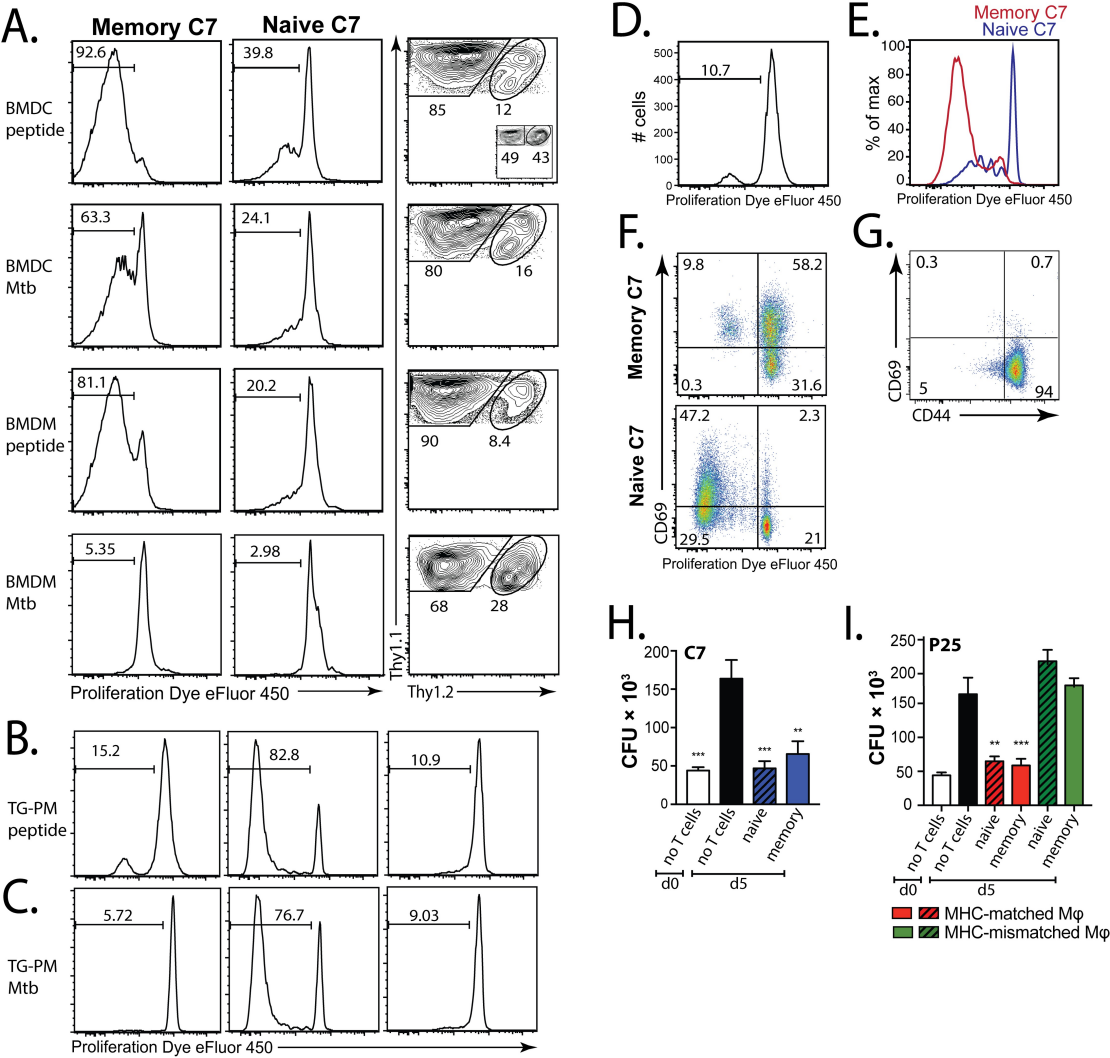
Mtb-infected DC, but not macrophages, stimulate memory CD4^+^ T cell proliferation. **(a)** eFluor450 dilution by memory (Thy1.1^+/+^, 1^st^ column) or naive (Thy1.1^+^/1.2^+^, 2^nd^ column) C7 cells, and their relative proportion (3^rd^ column) 4 d after co-culture with ESAT6_3-17_ peptide-coated BMDCs (1^st^ row), Mtb-infected BMDCs (2^nd^ row), peptide-coated BMDMs (3^rd^ row), or Mtb-infected BMDMs (4^th^ row). Inset (1^st^ row, 3^rd^ column) indicates input proportions of memory/naive C7 cells. eFluor450 dilution by memory (1^st^ column) and naive (2^nd^, 3^rd^ columns) C7 cells 5 d after co-culture with **(b)** peptide-loaded or **(c)** Mtb-infected, MHC-matched (C57BL10/J) thioglycolate-elicited peritoneal macrophages (TG-PMs) (1^st^, 2^nd^ columns), or MHC-mismatched (B10.BR) TG-PMs (3^rd^ columns). eFluor450 dilution by *in vitro* memory C7 cells 5d after culture with Mtb-infected **(d)** TG-PMs, or **(e)** BMDCs. **(f)** Representative CD69 and eFluor450 expression of *in vitro* memory (top) and naive (bottom) C7 cells 5 d after culture with Mtb-infected TG-PMs. **(g)** CD69 expression and eFluor450 dilution by vitro memory C7 cells prior to culture with TG-PMs. **(h, i)** Mtb-infected TG-PMs were cultured for 5 d alone or with (h) *in vitro* memory or naive C7 cells, or with **(i)** *in vitro* memory or naive P25 cells. The colony-forming units (CFU) were determined on the day of infection (d0) or on d5. As an additional control, *in vitro* memory or naive P25 cells were also cultured with MHC-mismatched (i.e., B10.BR TG-PMs).

An important principle of immunology is that naïve T cells are primed in the draining MLN by antigen-laden DCs [32]. As memory CD4^+^ T cells were first activated in the MLN and underwent robust proliferation (Fig 3A and 3E), but did not continue to expand in the lung (Fig 4A and 4B), we considered whether DCs and macrophages differ in their ability to stimulate memory and naive CD4^+^ T cell proliferation. Four days after culture with ESAT6_3-17_ peptide-loaded bone marrow-derived dendritic cells (BMDCs), memory C7 cells proliferated robustly (>90%), as did naïve C7 (40%) (Fig 6A, 1^st^ row), leading to their dominance (Fig 6A). When the same T cells were cultured with Mtb-infected BMDCs, memory C7 CD4^+^ T cells also divided more (>60%) compared to naïve C7 cells (20–25%), with a skewed ratio favoring the memory T cells (Fig 6A, 2^nd^ row). Peptide-coated bone marrow-derived macrophages (BMDMs) similarly induced proliferation in both memory and naïve C7 cells (>80% and 20%, respectively) (Fig 6A, 3^rd^ row). Importantly, neither memory nor naïve C7 cells proliferated when they were co-cultured with Mtb-infected BMDMs (Fig 6A, 4^th^ row). Although not surprising, these data indicate that Mtb-infected DCs stimulated better proliferation of CD4^+^ T cells than macrophages, and elicited more memory CD4^+^ T cell proliferation.

In addition to BMDMs, we tested thioglycolate-elicited peritoneal macrophages, which are more inflammatory, similar to macrophages that are recruited to the lung during TB. Five days after co-culture of memory and naïve ESAT6-specific C7 Tg CD4^+^ T cells with peptide-loaded inflammatory macrophages, only a small population of memory C7 cells proliferated (~15%), whereas naïve C7 cells proliferated robustly (>80%) (Fig 6B, left vs. middle), but not when co-cultured with MHC-mismatched macrophages (H-2^k^). (Fig 6B, right). Next, we co-cultured memory and naïve ESAT6-specific C7 Tg CD4^+^ T cells with Mtb-infected inflammatory macrophages. After five days, >75% of naïve C7 cells were proliferating (Fig 6C, middle), whereas few, if any, memory C7 cells had proliferated (Fig 6C, left). Neither naïve (Fig 6C, right) nor memory C7 cells proliferated when cultured with MHC-mismatched Mtb-infected inflammatory macrophages. Therefore, using inflammatory macrophages, we find that memory CD4^+^ T cells proliferate less efficiently than naïve CD4^+^ T cells.

We next sought to determine whether T cell proliferation and control of bacterial growth were linked. Since we were unable to obtain sufficient numbers of pure memory T cells from vaccinated mice, we generated *in vitro* memory C7 CD4^+^ T cells by stimulating naïve C7 cells once with ESAT6_3-17_-coated splenocytes in the presence of IL-2, IL-12, and anti-IL-4, as described [27,33,34]. After stimulation, these T cells were maintained in media supplemented with IL-2 and IL-7, and rested for 4 weeks. To validate these *in vitro* memory C7 CD4^+^ T cells, we cultured them with Mtb-infected inflammatory macrophages. As observed for vaccine-elicited memory C7 CD4^+^ T cells (Fig 6C), only 10% of the *in vitro* memory C7 CD4^+^ T cells proliferated (Fig 6D). Similarly, both *in vitro* memory and naïve C7 cells had proliferated 4d after co-culture with Mtb-infected BMDCs (Fig 6E). Despite their poor proliferation during culture with Mtb-infected macrophages, both memory and naïve CD4^+^ T cells specifically upregulated CD69, indicating activation (Fig 6F and 6G). Despite differences in proliferation, both naïve and memory C7 cells controlled Mtb growth in infected macrophages (Fig 6H). Similar experiments were done using naïve and *in vitro*-generated memory P25 Tg CD4^+^ T cells, which also controlled Mtb growth in MHC-matched, but not mismatched, inflammatory macrophages (Fig 6I). These data show that memory CD4^+^ T cells can exert potent effector function on Mtb-infected cells despite impaired proliferation.

### Memory CD4^+^ T cells protect early but not late after Mtb challenge

**Fig 7:**
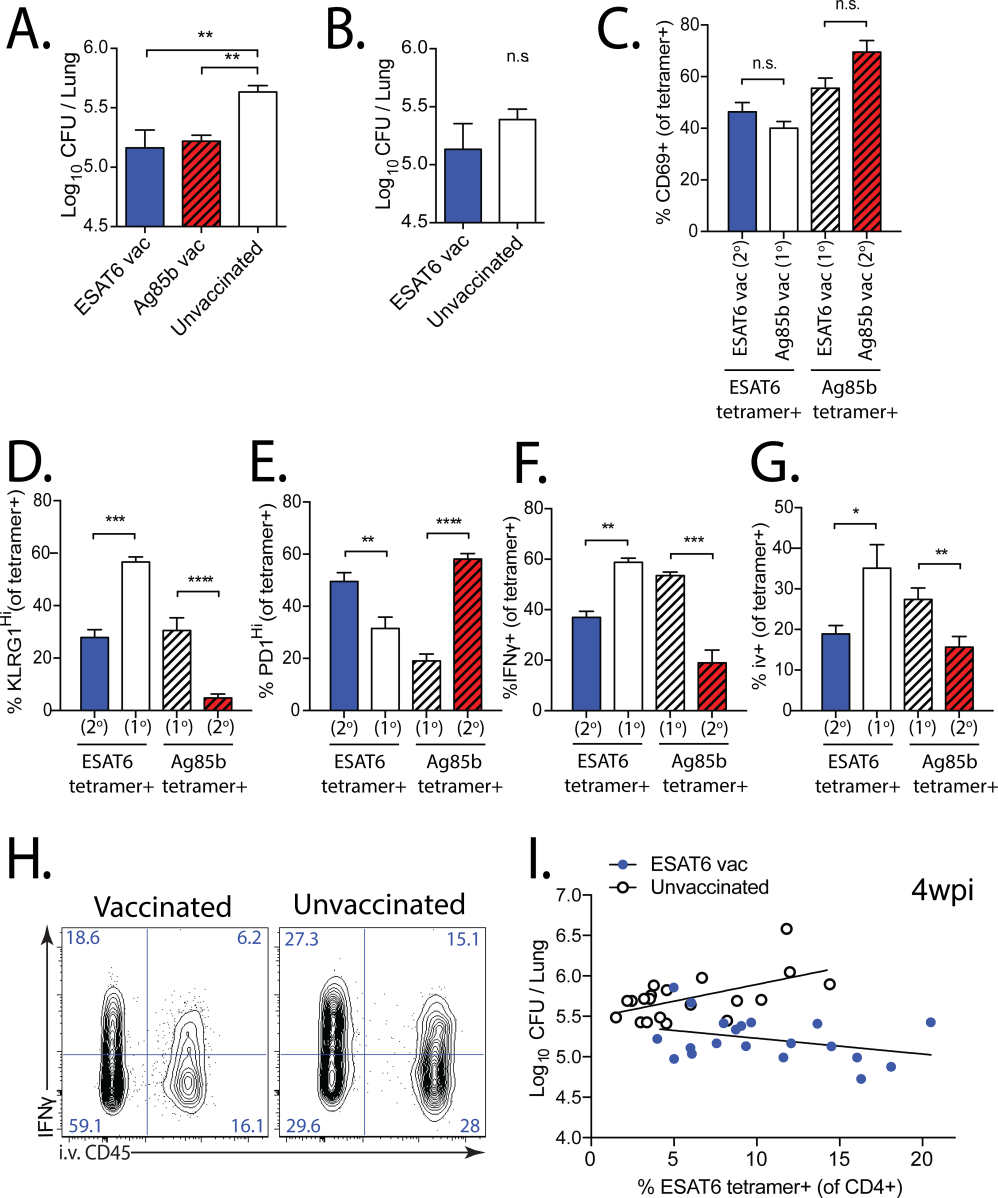
Memory CD4^+^ T cells are protective early, but not late, after Mtb challenge. **(a, b)** Mtb CFU quantified from the lungs of ESAT6- and Ag85b-vaccinated, or unvaccinated mice, 4 or 12 wpi. Expression of **(c)** CD69, **(d)** KLRG1, **(e)** PD-1, **(f)** IFNγ (unstimulated) by primary (1^o^) or secondary (2^o^) effector ESAT6_4-17_ and Ag85b_240-254_ tetramer^+^CD4^+^ T cells in the lungs of vaccinated mice 4 wpi. **(g)** Proportion of tetramer^+^CD4^+^ T cells in the lung intravascular compartment (ie. those that bind CD45 mAb injected i.v. 2 min prior to sacrifice) at 4wpi. **(h)** Representative plots of IFNγ expression and i.v. CD45 binding in the lung 4wpi in mice vaccinated with DDA-MPL ESAT6_3-17_ (left) or unvaccinated (right). **(i)** Paired CFU and ESAT6 tetramer frequency comparing ESAT6-vaccinated (blue) vs. unvaccinated (white) mice 4 wpi (from 4 experiments, each with n = 5/group). Non-linear regression with least squares fit was used to fit each group, after an extra sum of squares F-test determined that two different curves best fit the data (p<0.0001). R^2^ values for vaccinated and unvaccinated groups were 0.118 and 0.319, respectively.

To determine how memory T cell expansion affected the control of Mtb growth, we quantified the CD4^+^ T cell numbers and bacterial CFUs in the lungs of ESAT6 and Ag85b vaccinated mice after Mtb challenge. Four weeks after infection, a time point when the secondary responses in vaccinated mice were robust (Fig 1D), both ESAT6- and Ag85b-vaccinated mice had >0.5 log_10_ lower CFUs in the lung than unvaccinated mice (Fig 7A). However, by 12 wpi, when the frequency of ESAT6-specific CD4^+^ T cells was equivalent between ESAT6-vaccinated and unvaccinated mice (Fig 1G and 1H), there was no significant difference in bacterial load (Fig 7B), indicating that the protection conferred by vaccine-elicited memory CD4^+^ T cells is short-lived.

We next asked whether ESAT6 and Ag85b vaccination elicited memory CD4^+^ T cells with the potential to differentiate into secondary effector T cells that express a phenotype associated with protection against Mtb infection [35–38]. At 4 wpi, polyclonal ESAT6 and Ag85b-specific CD4^+^ T cell populations in vaccinated and unvaccinated mice had similar expression of CD69 (Fig 7C). Compared with the primary antigen-specific CD4^+^ T cell responses in unvaccinated mice, the secondary CD4^+^ T cell responses in ESAT6- and Ag85b-vaccinated mice contained fewer KLRG1^Hi^ and more PD1^Hi^ antigen-specific cells, were less likely to secrete IFNγ, and preferentially localized to the lung parenchymal compartment (i.v. CD45-) (Fig 7D–7G). While the primary CD4^+^ T cell responses were evaluated in both unvaccinated mice, as well as those vaccinated against a different antigen, for simplicity only the latter are displayed in the figure. Compared with unvaccinated mice, the decreased frequency of IFNγ-secreting ESAT6-specific CD4^+^ T cells at 4wpi in mice vaccinated with ESAT6 was ~50% greater among those that were “parenchymal” (i.v. CD45-) (20 ± 2.2% vs. 31 ± 5.4%; mean ± SEM), indicating that lower frequencies of IFNγ-secreting and i.v. CD45+ T cells were independent phenotypes of memory-derived T cell subsets (Fig 7H). To determine whether this phenotypic difference between vaccinated and unvaccinated mice persisted, we evaluated ESAT6-specific CD4^+^ T cells at 12 wpi, when no difference in bacterial load was observed between vaccinated vs. unvaccinated mice (Fig 7B). Despite similar CD69 expression, we found that differences in KLRG1 and PD-1 expression in antigen-specific CD4^+^ T cells did not significantly differ between vaccinated and unvaccinated mice (S7A–S7C Data). Interestingly, we detected overall fewer IFNγ-secreting ESAT6-specific CD4^+^ T cells in both groups, with vaccinated mice continuing to have slightly lower proportions of IFNγ-secreting and intravascular (i.v.+) cells than did unvaccinated mice (S7D and S7E Data).

To determine whether an increased frequency of ESAT6-specific CD4^+^ T cells was associated with greater protection in vaccinated mice, we pooled data from all the ESAT6-vaccinated vs. unvaccinated mice analyzed at 4 wpi (20 individual mice from 4 experiments). The correlation between the frequency of ESAT6-specific CD4^+^ T cells in the lung and CFU differed in vaccinated and unvaccinated mice (Fig 7I). In unvaccinated mice, we found that a higher frequency of ESAT6-specific CD4^+^ T cells was associated with higher bacterial load (Fig 7I). In vaccinated mice, however, the increased ESAT6-specific CD4^+^ T cell response was associated with a decrease in bacterial load (Fig 7I). However, at 12 wpi there was no significant difference in the CFU / ESAT6-specific response correlation between vaccinated and unvaccinated mice (S7F Data). We infer that in unvaccinated mice, the increased bacterial numbers, and therefore increased antigen, drive a greater T cell response, while in vaccinated mice, the increased numbers of memory-derived CD4^+^ T cells drive a lower bacterial load at 4 wpi, but not at 12 wpi when protection has waned in vaccinated mice. Therefore, we conclude that sustainable expansion and increased numbers of memory-derived CD4^+^ T cells late during infection would have the potential to prolong bacterial control.

## Discussion

In the current study, we use two models of vaccination to understand why memory CD4^+^ T cells elicit early, but not late, control of Mtb growth. In our ‘intact’ mouse model we elicit polyclonal memory CD4^+^ T cells specific for two distinct Mtb antigens, ESAT6 and Ag85b. Despite differences in the magnitude, TCRβ structure, and clonality of the responses to each antigen, memory CD4^+^ T cells specific for both ESAT6 and Ag85b reduced bacterial load early during TB. However, the accumulation of secondary (memory-derived) effector CD4^+^ T cells in vaccinated mice was slower than that of primary (naïve-derived) effector CD4^+^ T cells to each antigen. This effect was more dramatic for ESAT6, where 4–12 weeks after infection, no difference in the numbers of antigen-specific T cells in vaccinated vs. unvaccinated mice was detected. In vaccinated mice, both the relative increase in antigen-specific CD4^+^ T cells, and the relative decrease in bacterial load wane by later time points. Alternative explanations for the convergence of bacterial loads in vaccinated and unvaccinated mice late after Mtb challenge include preferential exhaustion of memory-derived CD4^+^ T cells, homeostatic regulatory mechanisms of host T cell numbers, including the effects of IFNγ, NO, and prostaglandin E2 (PGE2), or the development of bacterial resistance against memory T cell functions [28,35,39]. However, we did not observe differences in the expression of surface T cell exhaustion markers at 12 wpi. Furthermore, the concurrent equilibration of antigen-specific T cell numbers between vaccinated and unvaccinated mice, and the observation that greater antigen-specific T cell numbers in vaccinated mice is associated with greater protection (Fig 7I), makes the decreased expansion of memory-derived CD4^+^ T cells a likely cause of transient protection in the intact mouse model. Similarly, in our ‘transfer’ model, which controls for TCR and cell number differences, we observe that the primary response is more fit than the secondary response. Convergence of CD4^+^ T cell numbers between vaccinated and control mice during Mtb challenge has also been observed by others [37,40]. These data link the diminished expansion of the memory-derived CD4^+^ T cell response to the relative loss of Mtb control in vaccinated mice.

To determine why the rate of expansion differed between memory and naïve-derived CD4^+^ T cells, we compared the timing of T cell activation and differentiation, and the ability of memory vs. naive T cells to expand on a per-cell basis during infection. We discovered that both memory and naïve CD4^+^ T cells were activated first in the MLN after Mtb challenge, prior to their expansion in the lungs. Although our results do not exclude the possibility that tissue-resident memory (T_RM_) cells could localize to the lung and be triggered to proliferate after infection, neither the s.c. vaccination strategy nor the adoptive transfer of memory and naïve CD4^+^ T cells were designed to generate or study T_RM_ cells. While we observed a small number of eFluor450^Lo^ ESAT6-specific CD4^+^ cells in the lung at the d11 time point, this population (1) was similar for both naïve- and memory-derived CD4^+^ T cells, and (2) represented a tiny fraction of the total population, compared with an abundant population of dividing (eFluor450^Lo^) ESAT6-specific CD4^+^ T cells in the MLN at the same time point, indicating that they likely divided in the MLN and trafficked to the lung (Fig 3E). From these data, we conclude that both memory and naïve CD4^+^ T cells require initial activation in the MLN, a time at which similar proportions of naïve- and memory-derived T cells are dividing.

A comparison of the primary and secondary ESAT6-specific CD4^+^ T cells in the lungs showed similar activation, terminal differentiation and expression of T cell inhibitory receptors. However, once recruited to lung, the secondary responses to ESAT6 and Ag85b failed to expand as rapidly as the primary responses, and became outnumbered. Despite differences in their proliferation when co-cultured with Mtb-infected macrophages, both naïve and memory CD4^+^ T cells suppressed intracellular Mtb replication. The unique ability of Mtb-infected DCs to induce memory and naïve CD4^+^ T cell proliferation is consistent with the initial activation of these T cells in the LN after Mtb infection *in vivo*. The decreased uptake of EdU by memory-derived CD4^+^ T cells indicates that fewer of the cells in this population are proliferating in the lung at 4 wpi, compared with the primary response. However, we cannot exclude the possibility that memory-derived T cells undergo an increased rate of programmed cell death. In addition to activation-induced cell death, NO, PGE2, and IFNγ are abundant in the Mtb-infected lung and been shown to impair proliferation induce cell programmed cell death in T cells [35,39]. Unlike TB10.4-specific CD8^+^ T cells, we did not detect a gross difference in the sensitivity to antigen between memory vs. naïve C7 and P25 cells [23]. However, it is possible that a decreased sensitivity to antigen of does affect the resultant secondary vs. primary effectors, or other ESAT6- and Ag85b-specific CD4^+^ T cell clonotypes other than C7 and P25, to a greater extent.

Although the suboptimal stimulation of memory CD4^+^ T cells by Mtb-infected macrophages was striking, multiple factors affect memory T cell expansion *in vivo*. Two memory cell subsets have been defined based on their proliferative capacity, effector function, and location: central (T_CM_) and effector memory (T_EM_) [41]. Some have observed an association between vaccines that elicit T_CM_ and protection against TB [42]. Others promote the idea that T_EM_ and T_RM_ cells could respond directly to Mtb infection in the lung without the need for activation in secondary lymphoid organs [43]. Theoretically, by responding directly to infected cells in the lung, T_RM_ cells could accelerate the adaptive immune response to Mtb infection in vaccinated or previously-infected hosts. However, studies that generated memory T cells by prior aerosol Mtb infection and antibiotic clearance of Mtb found that the Mtb-specific CD4^+^ T cell response was accelerated only by 3–4 days in the lung and lymph nodes [8]. Using the same approach, we previously found no long-term difference in survival between these "memory mice" and naïve mice after Mtb challenge [5]. Despite eliciting a mixture of T_EM_ and T_CM_ by vaccination, we observed that T cell activation occurred first in the MLN with kinetics similar to the primary response. This does not mean that T_EM_ and T_RM_ cells couldn’t be initially activated in peripheral tissue. Instead, we predict that cognate interaction of memory T cells with Mtb-infected alveolar macrophages (AM) in the first days of infection is a rare event, given the large number of alveoli (~2 million per mouse) and a relatively low number of Mtb-specific T_RM_/T_EM_ T cells patrolling the lung. Thus, by concentrating and facilitating the interaction between Mtb-specific T cells and antigen-laden DCs, the LN may serve a crucial function for triggering the proliferation of both primary and secondary T cell responses. The cost of this increased efficiency is a delay in activating memory T cell responses.

Although Mtb elicits a strong T cell response, it is less certain whether the T cells are responding to infected cells or cells that have acquired bacterial antigen. Mounting evidence describes that Mtb impairs antigen presentation, which diminishes T cell recognition of infected cells [18,44–46]. For TB10.4-specific CD8^+^ T cells, the impaired expansion of memory T cells is in part due to a greater amount of antigen required to activate memory vs. naïve T cells, as previously observed in model systems [23,31]. We infer that there is a limited amount of TB10.4 antigen presented by in the lungs of infected mice. There is evidence for limited expansion of other antigen-specific memory CD8^+^ T cells (Rv3616), and a second epitope of TB10.4 [24]. In the current study, we observed only a small increase in the activation threshold of ESAT6-specific memory CD4^+^ T cells, and no difference in those specific for Ag85b. Therefore, the difference in proliferative potential of naïve and memory CD4^+^ T cells is likely to be due to factors other than the activation threshold.

IFNγ is critical to control of Mtb growth [10,15]. However, studies show that less-differentiated effector CD4^+^ T cells, even though they produce less IFNγ, are effective at mediating protection in part because they efficiently localize to the lung parenchyma [35,36,38]. Interestingly, the increased proportion of *parenchymal* Ag-specific CD4^+^ T cells in vaccinated mice 4wpi also contain fewer IFNγ-secreting cells, compared with unvaccinated mice, indicating that that these are independent protective characteristics enriched in memory-derived CD4^+^ T cells during TB (Fig 7H). We observed that vaccinated mice have lower Mtb CFUs and contain numerous antigen-specific CD4^+^ T cells expressing this protective phenotype. These data show that vaccination does elicit memory CD4^+^ T cells that, upon Mtb challenge, become activated, expand, localize to the lung parenchyma, and mediate protection. However, we infer that the inability of memory CD4^+^ T cells to sustainably expand limits their protective effect late during infection.

The impaired memory T cell expansion in the lungs during TB may diminish the efficacy of vaccines currently being clinically evaluated. BCG vaccination can elicit ~1 log_10_ protection in mice within 30 days after Mtb challenge. However, protection frequently diminishes over time [47,48]. Determining whether the transient nature of protection correlates with reduced memory cell fitness would require high quality immunological data over the course of infection. The H56 vaccine, a subunit vaccine consisting of the ESAT6, Ag85b, and Rv2660c proteins, leads to long-lasting CFU reduction [49]. An extrapolation from T cell cytokine expression data suggest the T cell numbers in BCG or H1 vaccinated mice declined during infection, and correlated with the loss of protection. A follow-up study reports early CFU control in H56 vaccinated mice [37]. Interestingly, the initial expansion of antigen-specific T cell numbers in the lungs of vaccinated mice returned to levels found in control mice by 4 wpi. Although we do not yet know whether the reduced fitness we observe for memory CD4^+^ T cells will apply to people, a BCG/H56 prime/boost strategy did not lead to persistent T cell immunity in the non-human primate model of TB, nor was significant protection observed [50].

Few reports have shown vaccine-elicited protection is sustained beyond 4–6 weeks after Mtb challenge. We believe that the lack of sustained proliferative responses by memory T cells in the infected lung limits the magnitude and duration of protection following vaccination. A major limitation to determining which vaccine candidates are likely to prevent TB in humans is the lack correlates of protection in pre-clinical vaccine studies. While at least two vaccines (BCG, MVA85A) show early protection in animal models of TB, none show reliable prevention of pulmonary disease in human efficacy trials [1,2]. Our data link the slower expansion of the memory-derived CD4^+^ T cell response after Mtb infection with the transient nature Mtb control in vaccinated mice. We conclude that the advantage conferred by many vaccine strategies fades over time, and protection is not durable. Given the dire need for an effective TB vaccine, we propose that a sustained increase in vaccine-elicited T cell numbers and long-lasting protection, facilitated by a proliferative response to Mtb-infected macrophages, should serve as benchmarks to determine which TB vaccine candidates should proceed to clinical trial.

## Materials and methods:

### Ethics Statement

The animal studies were approved by the Institutional Animal Care and Use Committee at the University of Massachusetts Medical School (Animal Welfare Assurance no. A3306-01), and adhere to the recommendations from the Guide for the Care and Use of Laboratory Animals of the National Institutes of Health and the Office of Laboratory Animal Welfare.

### Mice

B6 (C57BL/6J; CD45.2^+^Thy1.2^+^), CD45.1 (B6.SJL-Ptprc^a^Pepc^b^/BoyJ; CD45.1^+^Thy1.2^+^), Thy1.1 (B6.PL-Thy1^a^/CyJ; CD45.2^+^Thy1.1^+^), B10J (C57BL10/J), B10BR (B10.BR-H2k2 H2-T18a/SqSnJJrep) mice, and male P25 (C57BL/6-Tg(H2-K^b^-Tcrα,-Tcrβ)P25Ktk/J) mice for breeding, were all purchased from The Jackson Laboratories (Bar Harbor, ME). The P25 mice, expressing a TCR that is specific for Ag85b_240-254_ were bred on both the CD45.2^+^ and CD45.1^+^ congenic B6 backgrounds at the UMass Medical School animal facility. C7 TCR Tg mice, expressing a TCR specific for ESAT6_3-17_ on the Thy1.1 B6 background were donated by Dr. Eric Pamer (Memorial Sloan Kettering Cancer Center, NY). C7 mice were bred at UMass Medical School with Thy1.1 mice or Thy1.2 mice, resulting in C7 mice on both the Thy1.1 and Thy1.1/1.2 B6 backgrounds. All mice were housed under specific pathogen-free conditions. Mice were 7 to 12 weeks old at the start of all experiments. Mtb-infected mice were housed in biosafety level 3 facilities under specific pathogen-free conditions at UMass Medical School.

### Vaccination and assessment of immune responses

For DDA-TDM-MPL vaccination, 250μg of either ESAT6_3-17_ or Ag85b_240-254_ peptides were mixed with dimethyl dioctadecyl ammonium bromide (DDA) (Sigma). This mixture was emulsified with the adjuvant trehalose dicorynomycolate (TDM) + monophosphoryl lipid A (MPL) (Sigma), as described [20,51]. FQDAYNAAGGHNAVF (Ag85b_240-254_), EQQWNFAGIEAAASA (ESAT6_3-17_), and IMYNYPAM (TB10.4_4−11_), peptides were purchased from New England Peptides (Gardner, MA, USA) and reconstituted in DMSO (10mM). 200μl of this mixture was injected subcutaneously (s.c.) at the base of the neck of B6 mice. To generate memory C7 and P25 TCR Tg CD4^+^ T cells, 2x10^4^ naïve C7 or P25 cells were transferred to congenic (CD45.1^+^ or Thy1.1^+^) B6 mice, which were vaccinated the next day. In some experiments, peripheral blood T cell responses were monitored by flow cytometry, and compared with unvaccinated or control-vaccinated (TB10.4) mice. Mice were rested 12 weeks after a single vaccination to allow for the development of memory. Vaccination with peptide/Poly(I:C)/anti-CD40 was used to generate memory C7 or P25 TCR Tg T cells for some of the memory/naïve co-transfer experiments and for *in vitro* co-culture experiments, as this strategy generates greater numbers of memory CD4^+^ T cells [23,30]. Briefly, one day after adoptive transfer of 2x10^4^ naive C7 or P25 cells, mice were vaccinated intravenously with peptide/Poly(I:C)/anti-CD40 by tail vein injection and rested 12 weeks to generate memory. High molecular weight VacciGrade polyinosinic:polycytidylic acid [poly(I:C)] was obtained from InvivoGen (San Diego, CA), reconstituted in sterile PBS and stored at -20°C. Anti-CD40 mAb (clone FGK4.5) was purchased from BioXCell (West Lebanon, NH) and stored, undiluted, at -20°C. Vaccines were prepared by mixing 100 μmoles of either ESAT6_3-17_ or Ag85b_240-254_ peptide, 50 μg poly(I:C), and 50 μg anti-CD40 mAb, in a total volume of 200 μL with sterile PBS. A comparison of memory TCR Tg CD4^+^ T cells elicited by either vaccination strategy showed similar results (S6 Data). Co-transfer experiments were repeated with both vaccine strategies with equivalent results (S6b Data). Naïve C7 or P25^+^ TCR Tg CD4^+^ T cells were obtained from unvaccinated, age-matched TCR Tg mice rested for an equivalent period of time.

### Experimental infection and bacterial quantification

Mtb (strain Erdman) infections were performed via the aerosol route as described previously [13]. Infections were performed using a Glas-Col (Terre Haute, IN) full body inhalation exposure system. Mice received an inoculation dose of 25–75 CFU/mouse, as measured by plating undiluted lung homogenate within 24 hours of infection. At different times post-infection, mice were euthanized, organs were aseptically removed, individually homogenized in the FastPrep24 (MP Biomedicals, Santa Ana, CA, USA), and viable bacteria were enumerated by plating 10-fold serial dilutions of organ homogenates onto 7H11 agar plates (Hardy Diagnostics, Santa Maria, CA, USA). Mtb (strain H37Rv) *in vitro* infections of macrophages and DCs were performed as described previously [52]. H37Rv was grown and prepared as described [53]. Bacteria were counted in a Petroff-Hausser counter (Hausser Scientific, Horsham, PA, USA) and added to macrophages or DCs at an intended multiplicity of infection (MOI) of 5–10 for three hours. Cultures were washed three times to remove extracellular bacteria, and T cells were added the same day. For CFU measurement, cells were lysed with 1% Triton X-100/PBS and lysate from quadruplicate conditions on d0 and d5 post-infection, and 5-fold dilutions were plated on Middlebrook 7H11 agar plates (Hardy Diagnostics).

### Tissue and cell preparation

Lungs, MLNs, and spleens were removed after perfusion of the right ventricle with 10mL of cold RPMI1640 to purge the macrovasculature of the lungs. Lung cell suspensions were prepared by coarse dissociation using the GentleMACS tissue dissociator (Miltenyi Biotec, Germany). Lung tissue was digested for 30 min at 37°C with 250–300 U/mL Collagenase Type IV (Sigma) in complete RPMI1640 [10% heat-inactivated FCS (Sigma), 10 mM HEPES buffer, 1 mM sodium pyruvate, 2 mM L-glutamine, 10mM β-mercaptoethanol, 50 mg/ml streptomycin and 50 U/ml penicillin (all from Invitrogen)] followed by homogenization in the GentleMACS tissue dissociator and sequential straining through 70 μm and 40 μm nylon cell strainers (Falcon). Spleen and LN cell suspensions were prepared using gentle disruption of the organs through a 70 μm nylon strainer, followed by a 40 μm nylon cell strainer. For some experiments, erythrocytes were lysed in using ACK Lysis buffer (Sigma). For adoptive co-transfer experiments using naïve and memory TCR Tg CD4^+^ T cells, CD4^+^ T cells were enriched prior to surface antibody staining using either positive (Mouse CD4 T cell isolation kit, Miltenyi BIotec), or negative selection (EasySep Mouse CD4 T cell Isolation kit, StemCell Technologies, Vancouver, BC, Canada).

### Flow cytometric analysis

ESAT6_4-17_ and Ag85b_240-254_ I-A^b^ tetramers were obtained from the National Institutes of Health Tetramer Core Facility (Emory University Vaccine Center, Atlanta, GA, USA). Briefly, samples from Mtb-infected lung homogenates were resuspended in complete RPMI1640 (above), containing a 1:200 dilution of PE, APC, or BV421-conjugated MHC class II tetramers, and incubated for 1 hour at 37°C prior to antibody staining. Cell surface staining was performed with antibodies specific for mouse CD3ε (clone 145-2C11), CD4 (clone GK1.5), CD8 (clone 53–6.7), CD19 (clone 6D5), CD44 (clone IM7), CD45 (clone I3/2.3), CD62L (clone MEL-14), CD127 (clone A7R34), KLRG1 (clone 2F1/KLRG1), CXCR3 (clone CXCR3-173), CX3CR1 (clone SA011F11), PD-1 (clone 29F.1A12), 2B4 (clone m2B4 (B6)458.1), Lag-3 (clone C9B7W), CD69 (clone H1.2F3), CD25 (clone PC61), CD45.1 (clone A20), CD45.2 (clone 104), CD90.1 (clone OX-7), CD90.2 (clone 53–2.1), TCR Vβ11 (clone KT11) (all from Biolegend, San Diego, CA, USA), and TCR Vβ10(b) (clone B21.5) from BD Biosciences. Anti-Tim-3 (clone 5D12) was obtained from Dr. Vijay Kuchroo at Brigham and Women’s Hospital. IFNγ secretion was detected from T cells in the lung homogenate without restimulation using the IFNγ Cytokine Secretion Assay (Miltenyi Biotec), according to protocol. “Intravascular” vs. “parenchymal” antigen-specific CD4^+^ T cells in the lung were assayed in both the polyclonal and TCR Tg models, by injecting 2.5μg of fluorescently-labeled anti-CD45 (Biolegend) i.v. 2 min prior to sacrifice by CO_2_ asphyxiation, prior to perfusion with cold RPMI1640 by injection of the right ventricle, followed by tissue homgenization. A fixable, amine-reactive viability dye, Zombie Aqua (Biolegend), was used to exclude necrotic cells. All samples from Mtb-infected mice were fixed with 1% paraformaldehyde before analysis. Data were acquired using a MACSQuant flow cytometer (Miltenyi Biotec). Data were analyzed using FlowJo Software V9 (Tree Star, OR). For both analysis and cell sorting, single-lymphocyte events were gated by forward scatter area and height versus side scatter area for size and granularity.

### Adoptive T cell transfer of CD4^+^ T cells

Single cell suspensions of homogenized spleens and lymph nodes (inguinal, cervical, axillary, mediastinal, and mesenteric) were prepared from vaccinated mice containing memory C7 or P25 TCR Tg T cells (12 weeks after vaccination), or age-matched, unvaccinated C7 or P25 TCR Tg mice. CD4^+^ T cells were purified by negative selection using the EasySep Mouse CD4 T cell isolation kit (StemCell Technologies, Vancouver, BC, Canada), or the MojoSort Mouse CD4 T cell isolation kit (Biolegend), followed by magnetic separation. After enrichment, cells were stained with eFluor 450 proliferation dye (eBiosciences, USA), antibody-labeled and sorted by flow cytometry to achieve uniform populations of naïve or memory CD4^+^ T cells. For both C7 and P25 naïve/memory co-transfer experiments, 1x10^4^ cells of each population were mixed at a 1:1 ratio (confirmed by flow cytometry) and were transferred i.v. into congenic recipients (CD90.1 or CD45.1), which had been infected 6–7 d earlier with Mtb (strain Erdman). For priming experiments (Fig 3), 1–2×10^4^ memory and naïve C7 or P25 cells were co-transferred i.v. into mice infected with Mtb 6–7 d earlier. C7 TCR Tg CD4^+^ T cells used for the memory group were generated both on the Thy1.1, and Thy1.1/1.2 backgrounds to ensure that the congenic backgrounds of the mice did not influence the observed results. Similarly, memory P25 TCR Tg CD4^+^ T cells were generated on both the CD45.1^+^, and CD45.2^+^ backgrounds and were used in alternate co-transfer experiments. We additionally performed the co-transfer experiments without antibody labeling or flow-sorting, using flow cytometry analysis of a small sample of each population to mix naïve and memory Tg CD4^+^ T cells at a 1:1 ratio for injection into Mtb-challenged hosts, to ensure that differential antibody labeling did not preferentially affect either group, and observed similar results (S6C Data).

### Cell sorting

Fluorescent antibody-stained cells were flow-sorted using a FACS Aria II (Becton Dickinson) flow cytometer. For most adoptive co-transfer experiments using C7 TCR Tg CD4^+^ T cells, CD4^+^ CD8^-^ Vβ10^+^ Thy1.1^+^ KLRG1^Lo^ CD44^hi^ memory (from vaccinated mice containing C7 cells) or CD44^Lo^ naïve C7 cells (from age-matched, unvaccinated C7 mice) were sorted from pre-enriched CD4^+^ T cells. For co-transfer experiments using P25 TCR Tg CD4^+^ T cells, CD4^+^ CD8^-^ Vβ11^+^ CD45.1^+^ KLRG1^Lo^ CD44^hi^ memory, or CD45.2^+^ CD44^Lo^ naïve P25 cells were sorted. We additionally performed co-transfer experiments without antibody labeling or flow-sorting. For TCRβ repertoire analysis, we used duel-tetramer staining to identify and sort CD4^+^CD8^-^tetramer^+^CD44^Hi^ ESAT6- and Ag85b-specific CD4^+^ T cells, pre-enriched for CD4^+^ T cells from the lungs of B6 mice infected with Mtb Erdman 4–9 weeks earlier, as described [25]. For memory and secondary response experiments, we similarly sorted CD4^+^CD8^-^tetramer^+^CD44^Hi^ Ag85b-specific CD4^+^ T cells one week after vaccination with Ag85b_240-254_/poly(I:C)/anti-CD40 from 300–400 ul of peripheral blood. 12 weeks after vaccination, the same mice were challenged with Mtb, and three weeks later, CD4^+^CD8^-^tetramer^+^CD44^Hi^ T cells were sorted from the lungs, as described [23].

### Next generation sequencing

Genomic DNA was purified from sorted ESAT6_3-17_ and Ag85b_240-254_ tetramer^+^CD44^Hi^ CD4^+^ T cell populations using the QIAamp DNA Mini kit (Qiagen, Germany). High-throughput TCRβ sequencing was performed by Adaptive Biotechnologies Corp. (Seattle, WA, USA) (http://www.immunoseq.com) and analyzed using the ImmunoSEQ Analyzer toolset [54]. Clonality was calculated as entropy of the frequency distribution 1-(entropy/log_2_[# unique TCRs]) [55,56]. Transforming entropy in this manner results in a clonality score on a scale between 0–1. A score of “0” indicates that every TCR is unique; a score of “1” means that every TCR is the same. WebLogo 3 was used to identify CDR3β motifs (http://weblogo.threeplusone.com).

### Macrophage and DC in vitro culture

Bone marrow-derived dendritic cells (BMDCs) were prepared by culturing bone marrow cells (harvested from C57BL/6 mice) with complete RPMI1640, supplemented with 10 ng/mL granulocyte-macrophage colony stimulating factor (GM-CSF) (PeproTech, Rocky Hill, NJ, USA) for 8 days, as described [39]. Bone marrow-derived macrophages (BMDMs) were prepared by culturing bone marrow cells (from C57BL/6 mice) with complete RPMI1640, supplemented with 10% L929-conditioned media for 8 days, as described [57]. One day after plating, BMDCs and BMDMs were infected with Mtb, or incubated with 1 μM ESAT6_3-17_ peptide, followed by co-culture with T cells for 4 days. Thioglycolate-elicited peritoneal macrophages were prepared as described [52]. Briefly, the peritoneal cavity of B10/J or B10BR mice was lavaged 3–5 days after i.p. injection of a 3% thioglycolate solution. Anti-CD11b-conjugated microbeads (Miltenyi Biotec) were used to select CD11b^+^ macrophages from the lavage fluid. One day after plating, macrophages were infected with Mtb, and/or incubated with 1 μM ESAT6_3-17_ or Ag85b_240-254_ peptides, followed by co-culture with T cells for 3–5 days.

### Generation of in vitro memory CD4^+^ T cells

TCR Tg CD4^+^ T cells were isolated via negative selection (MojoSort CD4^+^ T cell isolation kit, Biolegend, San Diego, CA, USA). These TCR Tg CD4^+^ T cells (either C7 or P25) were cultured with irradiated B6 splenocytes (3200 Rads) (10^6^ T cells, 2x10^6^ splenocytes per well) with 5μM ESAT6_3-17_ or Ag85b_240-254_ peptides, IL-12 (10 ng/mL), IL-2 (100 U/mL, PeproTech), and anti-IL-4 mAb (1:500, Biolegend). Cells were split 2 days later, and media was supplemented with IL-2 and IL-7 (10U/mL and 5U/mL, PeproTech). Cells were split every 2–3 days for the first 10 days, followed by exchanges of half the volume of media with 2X media containing IL-2 and IL-7 twice weekly. After four weeks, in vitro memory T cells were used in co-culture experiments together with naïve C7 or P25 cells.

### Measurement of cell proliferation

The activation threshold of naïve vs. memory C7 or P25 TCR Tg CD4^+^ T cells was measured in vitro, as described [23]. Briefly, after memory and naïve (C7 or P25) TCR Tg CD4^+^ T cells were isolated from the spleens and lymph nodes, they were stained with 10 μM cell proliferation dye eFluor 450 (eBiosciences), and flow-sorted (as described above) to isolate pure populations of naïve or memory CD4^+^ T cells. Different concentrations of ESAT6_3-17_ or Ag85b_240-254_ peptide (serially diluted) were added to splenocytes from congenic CD45.1 or Thy1.1 B6 mice that served as APCs. The naïve and memory TCR Tg T cells were co-cultured with the APC at a 5:1:1 ratio (50,000 APCs, 5,000 memory and 5,000 naïve CD4^+^ T cells) in complete RPMI without cytokines. T cells and peptide-loaded splenocytes were co-incubated for 72-96h at 37°C. C7 and P25 cell proliferation was also measured after co-culture with peptide-laden splenocytes, or with macrophages or DCs infected with Mtb *in vitro* ± peptide. Proliferation was based on eFluor 450 dilution which was assessed by flow cytometry 4 days after co-culture with peptide-loaded APCs, or 4–5 days after co-culture with Mtb-infected macrophages or DCs ± peptide. To measure T cell proliferation in vivo, purified C7 or P25 TCR Tg CD4^+^ T cells were labeled with 10 μM of the cell proliferation dye eFluor 450. Analysis of C7 or P25 TCR Tg CD4^+^ T cell proliferation was measured after adoptive transfer into Mtb-infected mice, or into mice challenged with peptide/poly(I:C)/anti-CD40. Proliferation, determined by dye dilution, was measured by flow cytometry *in vivo* 11–12 days after aerosol Mtb infection. Cell proliferation at later time points (d21-22) *in vivo* was assayed by the incorporation of the synthetic thymidine analogue 5-Ethynyl-2’-deoxyuridine (EdU, Life Technologies). Briefly, 1 mg EdU diluted in 100 μL PBS was injected i.p. into each mouse 12h prior to analysis. After antibody staining, single cells suspensions were assayed for EdU incorporation using the Click-iT EdU Alexa Fluor 647 Flow Cytometry Assay kit (Life Technologies).

### Statistical analysis

All data are representative of 2–4 experiments, with 5 mice per group, unless stated otherwise. Data are represented as mean ± SEM. A two-tailed student’s t-test was used for normally-distributed data to compare two groups. When two groups were compared over multiple time points, two-tailed student’s t-tests were used for repeated measures. One-way or Two-way ANOVA were used to compare more than two groups, followed by Tukey post-tests. A p value < 0.05 was considered to be statistically significant. Statistical analyses were performed using Prism V7 (GraphPad Software, San Diego, CA).

## Supporting information

S1 Data Dominant clonotypes in the Ag8b-specific and ESAT6-specific CD4 T cell response to Mtb. The most abundant TCR from each sample of tetramer^+^ Ag8b-specific CD4 T cells (n = 8) and ESAT6-specific CD4 T cells (n = 10) is listed here and was used to generate Fig 2.
(PDF)

S2 Data TCR analysis of the Ag8b-specific and ESAT6-specific CD4 T cell response to Mtb. **(a)** The CDR3β amino sequence, the CDR3β length (left), TRBV (middle) and TRBJ (right) gene segment usage is shown for splenocytes from uninfected mice (top, n = 3), tetramer^+^Ag8b-specific CD4 T cells (middle, n = 8) and tetramer^+^ESAT6-specific CD4 T cells (bottom, n = 10). **(b)** The CDR3β amino acid sequence motifs “LEG” was identified among Ag8b-specific CD4 T cells that used Vβ16 with a CDR3β length of 36. The motif was derived from 82 unique DNA rearrangements accounting for 28 different TCRs (i.e., aa sequence). On average, these clonotypes accounted for 28% of the Ag85b-specific response, and were frequently expanded. **(c)** The CDR3β amino acid sequence motif “GG/TGG/GGG”, were identified among ESAT6-specific CD4 T cells using Vβ. These motifs are described in the text and in Fig 2. **(d)** Analysis of the CD4 T cell response to Ag85b, both after vaccination (e.g., in the blood), and after challenge (e.g., in the lung), is shown for TCRs using Vβ16 or non-Vβ16. The “LEG” motif was detected only among Vβ TCRs, both after vaccination and after Mtb challenge.
(TIF)

S3 Data Identification of the P25 and C7 TCRs in the polyclonal response. A. Description of transgenic TCRs. The transgenic TCRs used in this study. The P25-related and C7-related TCRs were closely related sequences detected in Mtb-infected mice, which had similar gene segment usage as P25 and C7, and closely related CDR3β sequences. P25 does not contain the ‘LEG’ motif that we frequently detected in Ag85b-specific CD4 T cells. C7 contains the ‘GGG’ motif that we observed in ESAT6-specific CD4 T cells. B. Detection of P25 TCR and related sequence in polyclonal response to Ag85b. The frequency of the P25 and P25-related CDR3β sequence in the tetramer^+^ Ag8b-specific CD4 T cells. Also listed are the frequency of TRBV16, TRBJ2-3, and TRBJ2-7, which are frequently used by Ag85b-specific CD4 T cells. Note that the P25 CDR3β amino acid sequence was not detected in any of our samples. C. Detection of C7 TCR and related sequence in polyclonal response to ESAT6. The frequency of the C7 and C7-related CDR3β amino acid sequence and number of unique clonotypes (based on DNA sequence) among the tetramer^+^ ESAT6-specific CD4 T cells sequenced.
(PDF)

S4 Data Early detection of the naïve and memory T cell responses in the lung. Gating strategy for whole MLN homogenate **(a, top left)** for P25 transfer experiments **(a, top row)**, or C7 transfer experiments **(a, bottom row)**. Representative plots of proliferation, CD62L and CD44 expression of P25 cells in the lung 12 days post-infection **(b).** Representative plots for C7 cells in the lung 11 days post-infection **(c).** For each, eFluor450 proliferation dye expression (left), and CD62L and CD44 expression are shown for dividing cells (middle) and non-dividing cells (right), in both memory-derived (top rows) and naïve-derived (bottom rows) CD4^+^ T cells. Data are representative of 2 independent experiments, each with 4 mice per group.
(TIF)

S5 Data Sequence of the C7 and P25 transgenic TCRs. 1a) C7 TCRα sequence. 1b) C7 TCRβ sequence. 2a) P25 TCRα sequence. 2b) P25 TCRβ sequence.
(PDF)

S6 Data Memory TCR Tg CD4^+^ T cells specific for ESAT6 (C7) generated by ESAT6 + DDA-TDM-MPL or Poly(I:C)/aCD40 vaccination exhibit similar impaired expansion in the lung after aerosol Mtb challenge. **(a)** Numbers of memory C7 cells in the lungs of separate groups of mice after adoptive transfer of C7 cells, vaccination with either DDA-TDM-MPL ESAT6 or Poly(I:C)/aCD40/ESAT6, and aerosol Mtb challenge 4 weeks earlier. **(b)** Proportions of memory vs. naive C7 cells at d15 post-infection, generated by either vaccine 12 weeks prior and co-transferred with naive C7 cells into the same mice. **(c)** Proportions of memory vs. naive C7 cells at 1 or 15 days after transfer into mice that were challenged with aerosol Mtb on d0. 1x10^4^ memory and naive C7 cells were co-transferred at a 1:1 ratio without the use of antibodies or flow sorting. n.s. not significant, **** <0.0001.
(PDF)

S7 Data Protective effects of CD4 vaccination are lost late after aerosol Mtb challenge. Expression of **(a)** CD69; **(b)** KLRG1; **(c)** PD-1; **(d)** IFNγ by ESAT6_4-17_ tetramer^+^CD4^+^ T cells in the lungs vaccinated (blue) or unvaccinated (white) mice 12 wpi. **(e)** Proportion of tetramer^+^CD4^+^ T cells in the lung intravascular (i.v. CD45+) compartment of ESAT6 vaccinated (blue) or unvaccinated (white) mice 12 wpi. **(f)** Paired CFU and ESAT6 tetramer frequency comparing ESAT6-vaccinated (blue) vs. unvaccinated (white) mice 12 wpi (from 3 experiments, each with n = 4-5/group). Non-linear regression with least squares fit was used to fit each group, after an extra sum of squares F-test determined that two different curves best fit the data (p = 0.0161). Runs test determined that the slopes were not significantly different from 0. R^2^ values for vaccinated and unvaccinated groups were 0.013 and 0.001, respectively.
(TIF)
