# Supplementary material for: Vaccine-elicited memory CD4+ T cell expansion is impaired in the lungs during tuberculosis
Source: PLoS Pathog. 2017 Nov 27;13(11):e1006704. doi: 10.1371/journal.ppat.1006704 (PMC5720822; doi:10.1371/journal.ppat.1006704)
Supplement: S1 Data — The most abundant TCR from each sample of tetramer+ Ag8b-specific CD4 T cells (n = 8) and ESAT6-specific CD4 T cells (n = 10) is listed here and was used to generate Fig 2. (PDF) [file ppat.1006704.s001.pdf]

Supplemental data 1.

Sample name	Productive frequency	Amino acid	CDR3β length	V gene	J gene
Ag85b_w4L1	20.2	CGARVTNSDYTF	12	TCRBV20-01	TCRBJ01-02
Ag85b_w4L2	17.5	CASSLEAETLYF	12	TCRBV16-01	TCRBJ02-03
Ag85b_w4L3	13.1	CASSLAANSDYTF	13	TCRBV03-01	TCRBJ01-02
Ag85b_w4L4	10.6	CASSLEFSNERLFF	42	TCRBV16-01	TCRBJ01-04
Ag85b_w4L5	17.9	CASS**LEG**DTQYF	14	TCRBV16-01	TCRBJ02-05
Ag85b_10w1	7.3	CASS**LEG**YEQYF	12	TCRBV16-01	TCRBJ02-07
Ag85b_10w2	19.1	CASS**LEG**NSDYTF	13	TCRBV16-01	TCRBJ01-02
Ag85b_10w3	14.9	CASSLEFSNERLFF	14	TCRBV16-01	TCRBJ01-04
ESAT6_w4L1	80.7	CASSPTVNNQAPLF	14	TCRBV26-01	TCRBJ01-05
ESAT6_w4L2	70.5	CASSR**TGG**YAETLYF	15	TCRBV26-01	TCRBJ02-03
ESAT6_w4L3	24.5	CASSIAGTKGAEQFF	15	TCRBV19-01	TCRBJ02-01
ESAT6_w4L4	51.3	CASSLS**TGG**PSDYTF	15	TCRBV26-01	TCRBJ01-02
ESAT6_w5L5	14.2	CASS**GGG**NTLYF	12	TCRBV13-01	TCRBJ02-04
ESAT6_10w1	52.3	CASSLW**TGG**KQNTLYF	16	TCRBV03-01	TCRBJ02-04
ESAT6_10w2	31.7	CASGVT**TGG**AEQFF	14	TCRBV13-02	TCRBJ02-01
ESAT6_10w3	43.6	CASKR**GG**SSAETLYF	15	TCRBV02-01	TCRBJ02-03
ESAT6_10w4	46.1	CASSPSNSGNTLYF	14	TCRBV19-01	TCRBJ01-03
ESAT6_10w5	57.3	CASSPHGQYSGNTLYF	16	TCRBV05-01	TCRBJ01-03

The most abundant TCR from each sample of tetramer^{+} Ag8b-specific CD4 T cells (n=8) and ESAT6-specific CD4 T cells (n=10) is listed here and was used to generate Fig.2. The CDR3β amino sequence, the CDR3β length, TRBV and TRBJ gene segment usage is shown. The “LEG” motif (which is detected in Ag8b-specific CD4 T cells) and “GG/TGG/GGG” motif (which is detected in ESAT6-specific CD4 T cells) is bolded. These motifs are described in the text and in Figure 2.
