# Supplementary figures and images for: Vaccine-elicited memory CD4+ T cell expansion is impaired in the lungs during tuberculosis
Source: PLoS Pathog. 2017 Nov 27;13(11):e1006704. doi: 10.1371/journal.ppat.1006704 (PMC5720822; doi:10.1371/journal.ppat.1006704)

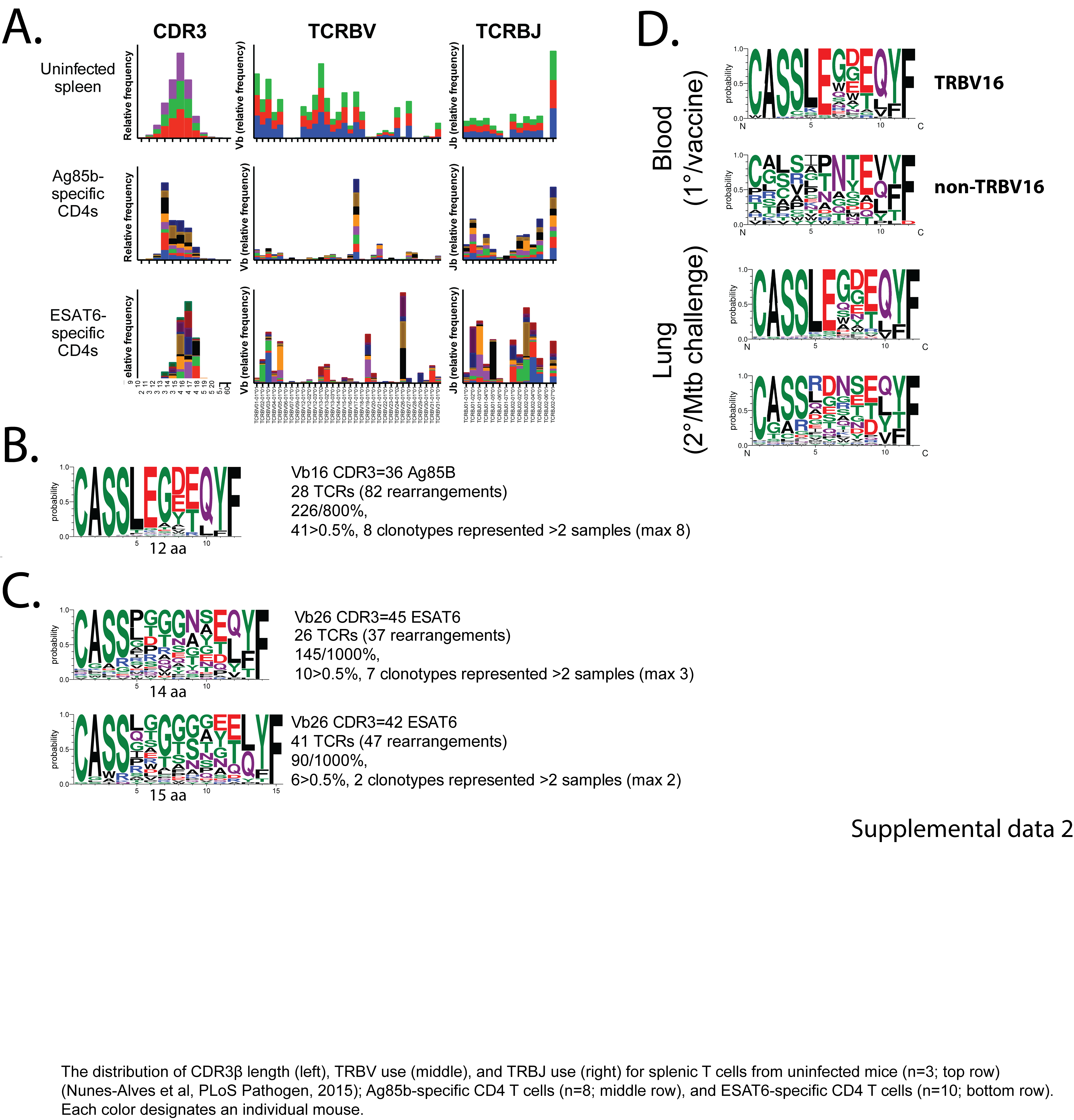

Supplement: S2 Data — (a) The CDR3β amino sequence, the CDR3β length (left), TRBV (middle) and TRBJ (right) gene segment usage is shown for splenocytes from uninfected mice (top, n = 3), tetramer+Ag8b-specific CD4 T cells (middle, n = 8) and tetramer+ESAT6-specific CD4 T cells (bottom, n = 10). (b) The CDR3β amino acid sequence motifs “LEG” was identified among Ag8b-specific CD4 T cells that used Vβ16 with a CDR3β length of 36. The motif was derived from 82 unique DNA rearrangements accounting for 28 different TCRs (i.e., aa sequence). On average, these clonotypes accounted for 28% of the Ag85b-specific response, and were frequently expanded. (c) The CDR3β amino acid sequence motif “GG/TGG/GGG”, were identified among ESAT6-specific CD4 T cells using Vβ. These motifs are described in the text and in Fig 2. (d) Analysis of the CD4 T cell response to Ag85b, both after vaccination (e.g., in the blood), and after challenge (e.g., in the lung), is shown for TCRs using Vβ16 or non-Vβ16. The “LEG” motif was detected only among Vβ TCRs, both after vaccination and after Mtb challenge. (TIF) [file ppat.1006704.s002.tif]

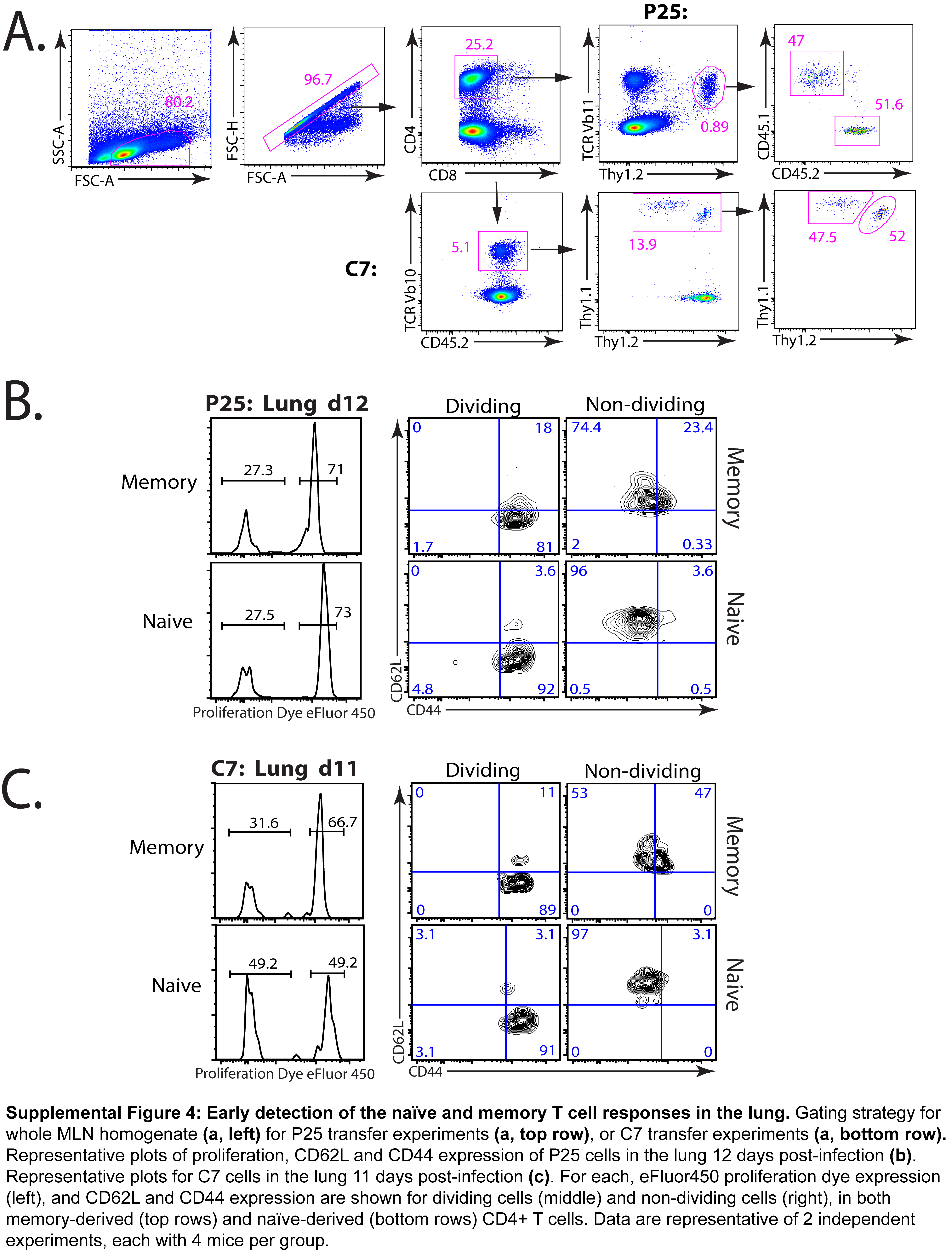

Supplement: S4 Data — Gating strategy for whole MLN homogenate (a, top left) for P25 transfer experiments (a, top row), or C7 transfer experiments (a, bottom row). Representative plots of proliferation, CD62L and CD44 expression of P25 cells in the lung 12 days post-infection (b). Representative plots for C7 cells in the lung 11 days post-infection (c). For each, eFluor450 proliferation dye expression (left), and CD62L and CD44 expression are shown for dividing cells (middle) and non-dividing cells (right), in both memory-derived (top rows) and naïve-derived (bottom rows) CD4+ T cells. Data are representative of 2 independent experiments, each with 4 mice per group. (TIF) [file ppat.1006704.s004.tif]

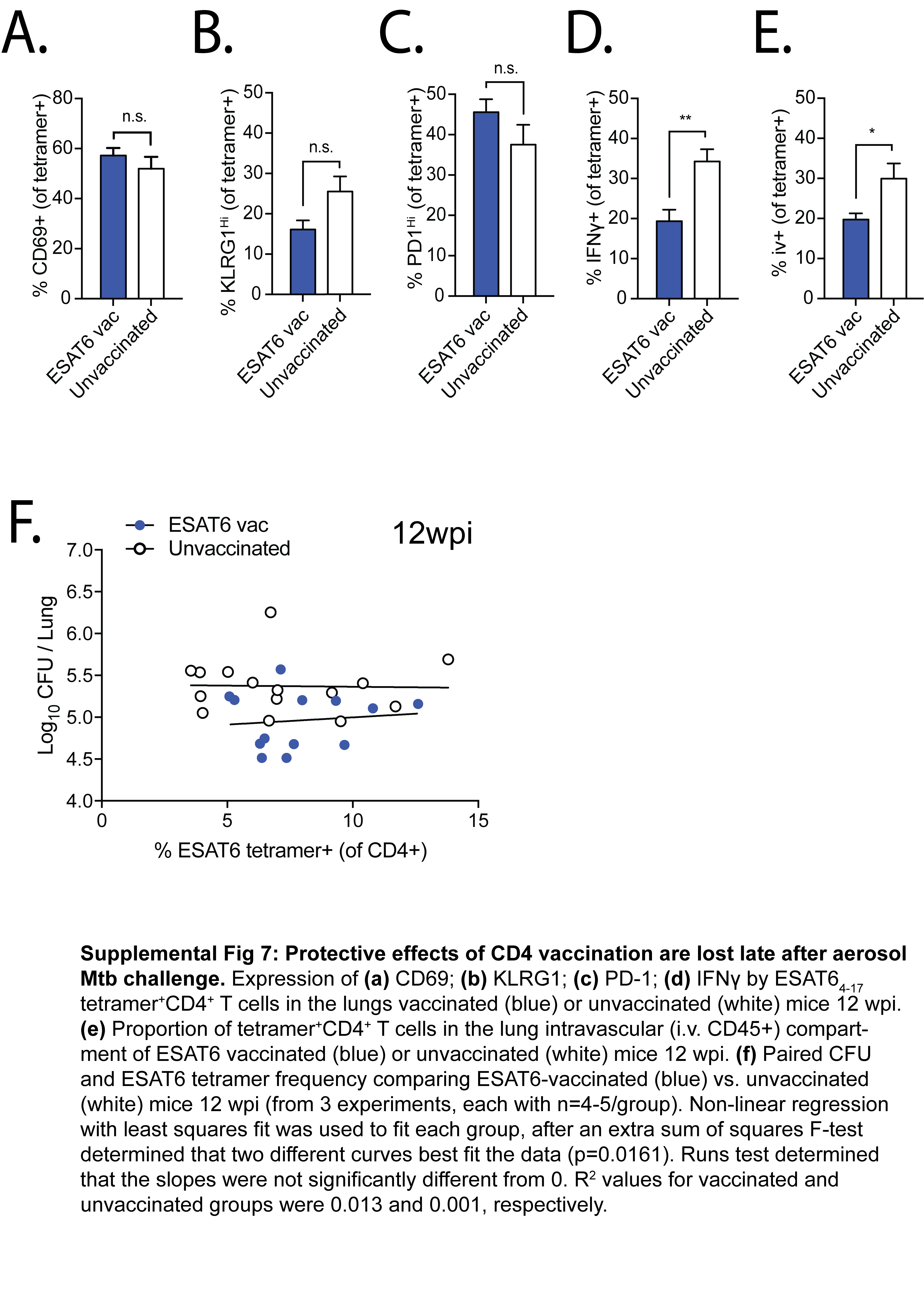

Supplement: S7 Data — Expression of (a) CD69; (b) KLRG1; (c) PD-1; (d) IFNγ by ESAT64-17 tetramer+CD4+ T cells in the lungs vaccinated (blue) or unvaccinated (white) mice 12 wpi. (e) Proportion of tetramer+CD4+ T cells in the lung intravascular (i.v. CD45+) compartment of ESAT6 vaccinated (blue) or unvaccinated (white) mice 12 wpi. (f) Paired CFU and ESAT6 tetramer frequency comparing ESAT6-vaccinated (blue) vs. unvaccinated (white) mice 12 wpi (from 3 experiments, each with n = 4-5/group). Non-linear regression with least squares fit was used to fit each group, after an extra sum of squares F-test determined that two different curves best fit the data (p = 0.0161). Runs test determined that the slopes were not significantly different from 0. R2 values for vaccinated and unvaccinated groups were 0.013 and 0.001, respectively. (TIF) [file ppat.1006704.s007.tif]
