# Supplementary material for: Vaccine-elicited memory CD4+ T cell expansion is impaired in the lungs during tuberculosis
Source: PLoS Pathog. 2017 Nov 27;13(11):e1006704. doi: 10.1371/journal.ppat.1006704 (PMC5720822; doi:10.1371/journal.ppat.1006704)
Supplement: S3 Data — A. Description of transgenic TCRs. The transgenic TCRs used in this study. The P25-related and C7-related TCRs were closely related sequences detected in Mtb-infected mice, which had similar gene segment usage as P25 and C7, and closely related CDR3β sequences. P25 does not contain the ‘LEG’ motif that we frequently detected in Ag85b-specific CD4 T cells. C7 contains the ‘GGG’ motif that we observed in ESAT6-specific CD4 T cells. B. Detection of P25 TCR and related sequence in polyclonal response to Ag85b. The frequency of the P25 and P25-related CDR3β sequence in the tetramer+ Ag8b-specific CD4 T cells. Also listed are the frequency of TRBV16, TRBJ2-3, and TRBJ2-7, which are frequently used by Ag85b-specific CD4 T cells. Note that the P25 CDR3β amino acid sequence was not detected in any of our samples. C. Detection of C7 TCR and related sequence in polyclonal response to ESAT6. The frequency of the C7 and C7-related CDR3β amino acid sequence and number of unique clonotypes (based on DNA sequence) among the tetramer+ ESAT6-specific CD4 T cells sequenced. (PDF) [file ppat.1006704.s003.pdf]

Supplemental Data 3

A. Description of transgenic TCRs

	P25	P25-related	C7 TCR	C7-related
Specificity	Ag85b	Ag85b	ESAT6	ESAT6
CDR3β sequence	CASSFESSAETLYF	CASSLESSAETLYF	CASSYWGGGQNTLYF	CASSSWGGGQNTLYF
TRBV	TCRBV16-01	TCRBV16-01	TCRBV04-01	TCRBV04-01
TRBJ	TCRBJ02-03	TCRBJ02-03	TCRBJ02-04	TCRBJ02-04
CDR3β length	14	14	15	15

B. Detection of P25 TCR and related sequence in polyclonal response to Ag85b.

Sample	P25	P25-related	TRBV16	TRBJ2-3	TRBJ2-7
Ag85b_10w1	None	0.12%	31.7%	3.0%	30.7%
Ag85b_10w2	None	None	34.7%	3.6%	29.3%
Ag85b_10w3	None	None	39.0%	4.6%	15.9%
Ag85b_w4L1	None	None	36.6%	11.6%	11.5%
Ag85b_w4L2	None	None	49.1%	27.4%	29.9%
Ag85b_w4L3	None	None	35.2%	None	17.9%
Ag85b_w4L4	None	None	59.8%	9.4%	30.1%
Ag85b_w4L5	None	4.73%	58.2%	7.9%	28.8%

C. Detection of C7 TCR and related sequence in polyclonal response to ESAT6.

	C7 TCR		C7-related	
Sample	frequency	Count	Row Labels	Count
ESAT6_10w1	0.0368%	1	None	0
ESAT6_10w2	None	0	None	0
ESAT6_10w3	0.0687%	1	None	0
ESAT6_10w4	0.0119%	1	0.229%	1
ESAT6_10w5	None	0	None	0
ESAT6_w4L1	None	0	None	0
ESAT6_w4L2	None	0	None	0
ESAT6_w4L3	None	0	None	0
ESAT6_w4L4	None	0	3.665%	1
ESAT6_w4L5	None	0	None	0

A. The transgenic TCRs used in this study. The P25-related and C7-related TCRs were closely related sequences detected in Mtb-infected mice, which had similar gene segment usage as P25 and C7, and closely related CDR3β sequences. P25 does not contain the 'LEG' motif that we frequently detected in Ag85b-specific CD4 T cells. C7 contains the 'GGG' motif that we observed in ESAT6-specific CD4 T cells.

B. The frequency of the P25 and P25-related CDR3β sequence in the tetramer^{+}Ag8b-specific CD4 T cells. Also listed are the frequency of TRBV16, TRBJ2-3, and TRBJ2-7, which are frequently used by Ag85b-specific CD4 T cells. Note that the P25 CDR3β amino acid sequence was not detected in any of our samples.

C. The frequency of the C7 and C7-related CDR3β amino acid sequence and number of unique clonotypes (based on DNA sequence) among the tetramer^{+}ESAT6-specific CD4 T cells sequenced.
