# Supplementary material for: Vaccine-elicited memory CD4+ T cell expansion is impaired in the lungs during tuberculosis
Source: PLoS Pathog. 2017 Nov 27;13(11):e1006704. doi: 10.1371/journal.ppat.1006704 (PMC5720822; doi:10.1371/journal.ppat.1006704)
Supplement: S5 Data — 1a) C7 TCRα sequence. 1b) C7 TCRβ sequence. 2a) P25 TCRα sequence. 2b) P25 TCRβ sequence. (PDF) [file ppat.1006704.s005.pdf]

Supplemental Data 5.

1) C7 TCR sequence

IMGT analysis:	TRA rearranged sequence	TRB rearranged sequence
**V-GENE and allele**	Musmus TRAV4D-4*02 (F)	Musmus TRBV4*01 F
**D-GENE and allele**	N/A	Musmus TRBD2*01 F
**J-GENE and allele**	Musmus TRAJ34*02 F	Musmus TRBJ2-4*01 F
**AA JUNCTION**	CAAGNTNKVVF	CASSYWGGGQNTLYF

1a) C7 TCRalpha sequence

ATGCAGAGGAACCTGGGAGCTGTGCTGGGGATTCTGTGGGTGCAGATTTGCTGGGTGAGAGGAGATCAGGTGGA
GCAGAGTCCTTCAGCCCTGAGCCTCCACGAGGGAACCGGTTCTGCTCTGAGATGCAATTTTACGACCACCATGA
GGGCTGTGCAGTGGTTCCAACAGAACTCCAGGGGCAGCCTCATCAATCTGTTCTACTTGGCTTCAGGAACAAAG
GAGAATGGAGGTTAAAGTCAACATTCAATTCTAAGGAGAGCTACAGCACCCTGCACATCAGGGATGCCCAGCT
GGAGGACTCAGGCACTTACTTCTGT**GCTGCTGGGAATACCAACAAAGTCGTC**TTTGGAACAGGGACCAGATTAC
AAGTATTACCAA*ACATCCAGAATCCGGAACCTGCTGTGTACCAGTTAAAAGATCCTCGGTCTCAGGACAGCACC*
CTCTGCCTGTTCACCGACTTTGACTCCCAAATCAATGTGCCGAAAACCATGGAATCTGGAACGTTCATCACTGA
CAAAACTGTGCTGGACATGAAAGCTATGGATTCCAAGAGCAATGGGGCCATTGCCTGGAGCAACCAGACAAGCT
TCACCTGCCAAGATATCTTCAAAGAGACCAACGCCACCTACCCCAGTTCAGACGTTCCCTGTGATGCCACGTTG
ACTGAGAAAAGCTTTGAAACAGATATGAACCTAAACTTTCAAAACCTGTCAGTTATGGGACTCCGAATCCTCCT
GCTGAAAGTAGCCGGATTTAACCTGCTCATGACGCTGAGGCTGTGGTCC

1b) C7 TCRbeta sequence

ATGGGCTGTAGGCTCCTAAGCTGTGTGGCCTTCTGCCTCTTGGGAATAGGCCCTTTGGAGACGGCTGTTTTCCA
GACTCCAAACTATCATGTCACACAGGTGGGAAATGAAGTGTCTTTCAATTGTAAGCAAACTCTGGGCCACGATA
CTATGTATTGGTACAAGCAAGACTCTAAGAAATTGCTGAAGATTATGTTTAGCTACAATAATAAGCAACTCATT
GTAAACGAAACAGTTCCAAGGCGCTTCTCACCTCAGTCTTCAGATAAAGCTCATTTGAATCTTCGAATCAAGTC
TGTAGAGCCGGAGGACTCTGCTGTGTATCTCTGT**GCCAGCAGCTATTGGGGGGGCGGTCAAAACACCTTGTAC**T
TTGGTGCGGGCACCCGACTATCGGTGCTAG*AAGATCTGAGAAATGTGACTCCACCCAAGGTCTCCTTGTTTGAG*
CCATCAAAAGCAGAGATTGCAAACAAACAAAAGGCTACCCTCGTGTGCTTGGCCAGGGGCTTCTTCCCTGACCA
CGTGGAGCTGAGCTGGTGGGTGAATGGCAAGGAGGTCCACAGTGGGGTCAGCACGGACCCTCAGGCCTACAAGG
AGAGCAATTATAGCTACTGCCTGAGCAGCCGCCTGAGGGTCTCTGCTACCTTCTGGCACAATCCTCGAAACCAC
TTCCGCTGCCAAGTGCAGTTCCATGGGCTTTCAGAGGAGGACAAGTGGCCAGAGGGCTCACCCAAACCTGTCAC
ACAGAACATCAGTGCAGAGGCCTGGGGCCGAGCAGACTGTGGAATCACTTCAGCATCCTATCATCAGGGGGTTC
TGTCTGCAACCATCCTCTATGAGATCCTACTGGGGAAGGCCACCCTATATGCTGTGCTGGTCAGTGGCCTGGTG
CTGATGGCCATGGTCAAGAAAAAAAATTCCTGA

2) P25 TCR sequence

IMGT analysis:	TRA rearranged sequence	TRB rearranged sequence
**V-GENE and allele**	Musmus TRAV3D-3*02 F	Musmus TRBV16*01 F
**D-GENE and allele**	N/A	Musmus TRBD1*01 F
**J-GENE and allele**	Musmus TRAJ18*01 F	Musmus TRBJ2-3*01 F
**AA JUNCTION**	CAVSGGSALGRLHF	CASSFESSAETLYF

2a) P25 TCRalpha sequence

ATGAAGACAGTGACTGGACCTTTGTTCCTGTGCTTCTGGCTGCAGCTGAACTGTGTGAGCAGAGGCGAGCAGGT
GGAGCAGCGCCCTCCTCACCTGAGTGTCCGGGAGGGAGACAGTGCCGTTATCATCTGCACCTACACAGACCCTA
ACAGTTATTACTTCTTCTGGTACAAGCAAGAGCCGGGGGCAGGTCTTCAGTTGCTTATGAAGGTTTTCTCAAGT
ACGGAAATAAACGAAGGACAAGGATTCACTGTCCTACTGAACAAGAAAGACAAACAACTCTCTCTGAACCTCAC
AGCTGCCCATCCTGGGGACTCAGCCGTGTACTTCTGCGCAGTCAGTGGAGGTTCAGCCTTAGGGAGGCTGCATT
TTGGAGCTGGGACTCAGCTGATTGTCATACCTGACATCCAGAACCCAGAACCTGCTGTGTACCAGTTAAAAGAT

CCTCGGTCTCAGGACAGCACCCTCTGCCTGTTCACCGACTTTGACTCCCAAATCAATGTGCCGAAAACCATGGA
ATCTGGA*ACGTTCATCACTGACAAAACTGTGCTGGACATGAAAGCTATGGATTCCAAGAGCAATGGGGCCATTG*
CCTGGAGCAACCAGACAAGCTTCACCTGCCAAGATATCTTCAAAGAGACCAACGCCACCTACCCCAGTTCAGAC
GTTCCCTGTGATGCCACGTTGACTGAGAAAAGCTTTGAAACAGATATGAACCTAAACTTTCAAAACCTGTCAGT
TATGGGACTCCGAATCCTCCTGCTGAAAGTAGCCGGATTTAACCTGCTCATGACGCTGAGGCTGTGGTCCAGTT
GAGGTCTGCAAGGATCC

2b) P25 TCRbeta sequence
ATGGCCCCCAGGCTCCTTTTCTGTCTGGTTCTTTGCTTCTTGAGAGCAGAACCAACAAATGCTGGTGTCATCCA
AACACCTAGGCACAAGGTGACAGGGAAGGGACAAGAAGCAACTCTGTGGTGTGAGCCAATTTCAGGACATAGTG
CTGTTTTCTGGTACAGACAGACCATTGTGCAGGGCCTGGAGTTCCTGACTTACTTTCGAAATCAAGCTCCTATA
GATGATTCAGGGATGCCCAAGGAACGATTCTCAGCTCAGATGCCCAATCAGTCGCACTCAACTCTGAAGATCCA
GAGCACGCAACCCCAGGACTCAGCGGTGTATCTTTGTGCAAGCAGCTTCGAATCTAGTGCAGAAACGCTGTATT
TTGGCTCAGGAACCAGACTGACTGTTCTCG*AGGATCTGAGAAATGTGACTCCACCCAAGGTCTCCTTGTTTGAG*
CCATCAAAAGCAGAGATTGCAAACAAACAAAAGGCTACCCTCGTGTGCTTGGCCAGGGGCTTCTTCCCTGACCA
CGTGGAGCTGAGCTGGTGGGTGAATGGCAAGGAGGTCCACAGTGGGGTCAGCACGGACCCTCAGGCCTACAAGG
AGAGCAATTATAGCTACTGCCTGAGCAGCCGCCTGAGGGTCTCTGCTACCTTCTGGCACAATCCTCGAAACCAC
TTCCGCTGCCAAGTGCAGTTCCATGGGCTTTCAGAGGAGGACAAGTGGCCAGAGGGCTCACCCAAACCTGTCAC
ACAGAACATCAGTGCAGAGGCCTGGGGCCGAGCAGACTGTGGAATCACTTCAGCATCCTATCATCAGGGGGTTC
TGTCTGCAACCATCCTCTATGAGATCCTACTGGGGAAGGCCACCCTATATGCTGTGCTGGTCAGTGGCCTGGTG
CTGATGGCCATGGTCAAGAAAAAAAATTCCTGAGACAAACTTTTATGC

Code:
V region (single underline)
J region (double underline)
D region (turquoise highlight)
N or P region (yellow highlight)
CDR3 (bold)
C region (italics)
Green highlight – difference from germline (supposed to be “T”)
