# Supplementary material for: Vaccine-elicited memory CD4+ T cell expansion is impaired in the lungs during tuberculosis
Source: PLoS Pathog. 2017 Nov 27;13(11):e1006704. doi: 10.1371/journal.ppat.1006704 (PMC5720822; doi:10.1371/journal.ppat.1006704)
Supplement: S6 Data — (a) Numbers of memory C7 cells in the lungs of separate groups of mice after adoptive transfer of C7 cells, vaccination with either DDA-TDM-MPL ESAT6 or Poly(I:C)/aCD40/ESAT6, and aerosol Mtb challenge 4 weeks earlier. (b) Proportions of memory vs. naive C7 cells at d15 post-infection, generated by either vaccine 12 weeks prior and co-transferred with naive C7 cells into the same mice. (c) Proportions of memory vs. naive C7 cells at 1 or 15 days after transfer into mice that were challenged with aerosol Mtb on d0. 1x10^4^ memory and naive C7 cells were co-transferred at a 1:1 ratio without the use of antibodies or flow sorting. n.s. not significant, **** <0.0001. (PDF) [file ppat.1006704.s006.pdf]

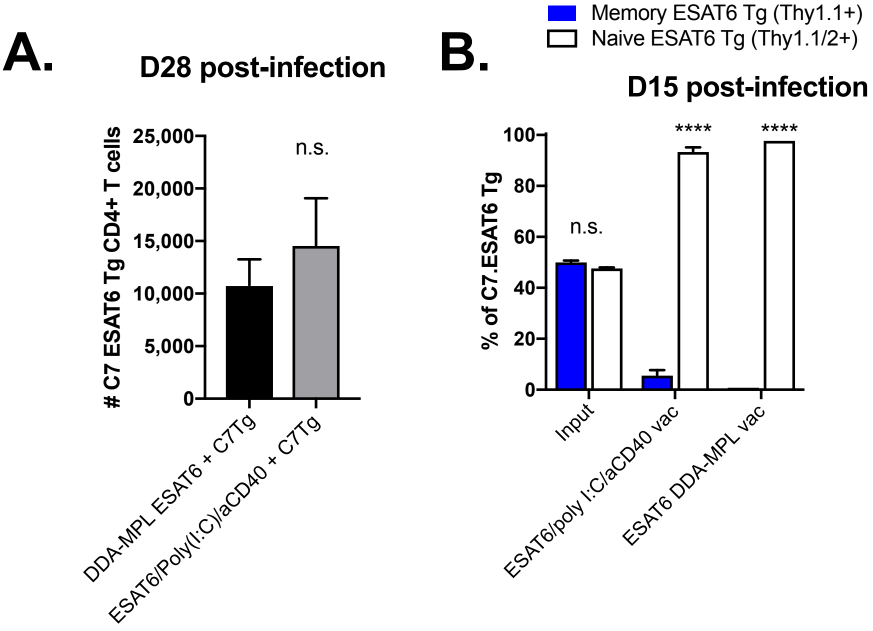

Fig. S6: Memory TCR Tg CD4+ T cells specific for ESAT6 (C7) generated by ESAT6 + DDA-TDM-MPL or Poly(I:C)/aCD40 vaccination exibit similar impaired expansion in the lung after aerosol Mtb challenge. (a) Numbers of memory C7 cells in the lungs of separate groups of mice after adoptive transfer of C7 cells, vaccination with either DDA-TDM-MPL ESAT6 or Poly(I:C)/aCD40/ESAT6, and aerosol Mtb challege 4 weeks earlier.
(b) Proportions of memory vs. naive C7 cells at d15 post-infection, generated by either vaccine 12 weeks prior and co-transferred with naive C7 cells into the same mice. n.s. not significant, **** <0.0001.

Supplemental Fig.6
